# Polychlorinated biphenyls (PCBs) as sentinels for the elucidation of Arctic environmental change processes: a comprehensive review combined with ArcRisk project results

**DOI:** 10.1007/s11356-018-2625-7

**Published:** 2018-06-28

**Authors:** Pernilla Carlsson, Knut Breivik, Eva Brorström-Lundén, Ian Cousins, Jesper Christensen, Joan O. Grimalt, Crispin Halsall, Roland Kallenborn, Khaled Abass, Gerhard Lammel, John Munthe, Matthew MacLeod, Jon Øyvind Odland, Janet Pawlak, Arja Rautio, Lars-Otto Reiersen, Martin Schlabach, Irene Stemmler, Simon Wilson, Henry Wöhrnschimmel

**Affiliations:** 1 Norwegian Institute for Water Research (NIVA), 0349 Oslo, Norway; 2 NILU—Norwegian Institute for Air Research, 2027 Kjeller, Norway; 3 IVL Swedish Environment Research Institute, 411 33 Göteborg, Sweden; 4 Department of Environmental Science and Analytical Chemistry (ACES), Stockholm University, 11418 Stockholm, Sweden; 5 Department of Bioscience, Arctic Research Centre, Aarhus University, 4000 Roskilde, Denmark; 6 Institute of Environmental Assessment and Water Research (IDÆA), Spanish Council for Scientific Research (CSIC), 0834 Barcelona, Spain; 7 Lancaster Environment Centre, Lancaster University, Lancaster, LA1 4YQ UK; 8 Faculty of Chemistry, Biotechnology and Food Sciences (KBM), Norwegian University of Life Sciences (NMBU), Christian Magnus Falsen Veg 1, 1432 Ås, Norway; 9 Department of Arctic Technology (AT), University Centre in Svalbard (UNIS), 9171 Longyearbyen, Svalbard Norway; 10 Department of Pesticides, Menoufia University, P.O. Box 32511, Shebeen El-Kom, Egypt; 11 Arctic Health, Faculty of Medicine, University of Oulu, 90014 Oulu, Finland; 12 Max Planck Institute for Chemistry, 55128 Mainz, Germany; 13 Research Centre for Toxic Compounds in the Environment, Masaryk University, 62500 Brno, Czech Republic; 14 Department of Community Medicine, UiT—The Arctic University of Norway, 9037 Tromsø, Norway; 15 Arctic Monitoring and Assessment Programme (AMAP), AMAP Secretariat, Gaustadalléen 21, 0349 Oslo, Norway; 16 Max Planck Institute for Meteorology, 20146 Hamburg, Germany; 17 Department of Chemistry and Applied Biosciences, Institute of Chemical and Bioengineering, ETH Zürich, 8092 Zürich, Switzerland; 18 Present Address: Swiss Federal Office for the Environment, Worblentalstrasse 68, 3063 Ittigen, Switzerland

**Keywords:** Polychlorinated biphenyls, PCB, Arctic, Climate change, Environmental properties, Distribution pathways, Environmental fate

## Abstract

Polychlorinated biphenyls (PCBs) can be used as chemical sentinels for the assessment of anthropogenic influences on Arctic environmental change. We present an overview of studies on PCBs in the Arctic and combine these with the findings from *ArcRisk*—a major European Union-funded project aimed at examining the effects of climate change on the transport of contaminants to and their behaviour of in the Arctic—to provide a case study on the behaviour and impact of PCBs over time in the Arctic. PCBs in the Arctic have shown declining trends in the environment over the last few decades. Atmospheric long-range transport from secondary and primary sources is the major input of PCBs to the Arctic region. Modelling of the atmospheric PCB composition and behaviour showed some increases in environmental concentrations in a warmer Arctic, but the general decline in PCB levels is still the most prominent feature. ‘Within-Arctic’ processing of PCBs will be affected by climate change-related processes such as changing wet deposition. These in turn will influence biological exposure and uptake of PCBs. The pan-Arctic rivers draining large Arctic/sub-Arctic catchments provide a significant source of PCBs to the Arctic Ocean, although changes in hydrology/sediment transport combined with a changing marine environment remain areas of uncertainty with regard to PCB fate. Indirect effects of climate change on human exposure, such as a changing diet will influence and possibly reduce PCB exposure for indigenous peoples. Body burdens of PCBs have declined since the 1980s and are predicted to decline further.

## Background

The Arctic environment and ecosystems are changing. For example, the Arctic cryosphere—the major feature of the marine and terrestrial Arctic—is undergoing considerable change (Olsen et al. 2011) with the marine ice cover during the Arctic summer recently reaching a record minimum extent in 2012 (AMAP 2017). As a result, the ice-associated ecosystems in the Arctic are under increasing pressure (Grannas et al. 2013). The marine pelagic and benthic food webs are changing and new invasive species are competing with native Arctic species for food sources (Renaud et al. 2012). Fish stocks previously observed exclusively in lower latitude waters are moving into the Arctic marine environment for spawning (Kallenborn et al. 2012). These significant environmental changes are also expected to influence directly or indirectly the distribution patterns and fate of persistent organic pollutants (POPs) in the Arctic environment (AMAP 2003; AMAP 2011; UNEP/AMAP 2011; Kallenborn et al. 2012; Macdonald and Bewers 1996; Macdonald et al. 2003; Macdonald et al. 2005; Pucko et al. 2015).

POPs are among the most investigated anthropogenic pollutants in the global environment. Their impact and hazardous effects on biotic and abiotic structures of the world’s ecosystems have been documented in thousands of scientific studies and reports, and the risks of POPs have been well known for half a century already (Baldassare and Nicolle 1989; Ballschmiter and Zell 1980; Bowes and Jonkel 1972; Bright et al. 1995; Carpenter 1998; Jensen et al. 1969; Jones 1988; Lang 1992; McKinney and Waller 1994; Tanabe et al. 1983; Zitko and Choi 1972). Several early temporal trend studies revealed that Northern ecosystems have been exposed to POPs since the 1960–1970s (Bignert et al. 1998; Braune et al. 2001; Braune and Simon 2003). In the 1980s, combined field and modelling studies confirmed that semi-volatile POPs are transported into the Arctic via a combination of oceanic and atmospheric transport pathways (Barrie et al. 1992; Bidleman 1988; Wania and Mackay 1993, 1995, 1996). The strong bioaccumulation potential of these pollutants, to which Arctic food webs had already been exposed for several decades, leads to documented high POP concentrations in lipid tissues of Arctic marine mammals (Braune et al. 2005; Hickie et al. 2005; Ikonomou and Addison 2008; Kucklick et al. 2002). Marine mammals are the major traditional food source for indigenous Arctic peoples who rely on the availability and high nutritional values of this meat (Sharma 2010). Consequently, from some of the first studies examining POPs in humans living in the Arctic, elevated POP levels were identified in Canadian and Greenlandic Inuit living according to their traditional culture, which includes seasonal hunting of marine mammals (Bonefeld-Jørgensen 2010; Bonefeld-Jørgensen and Long 2010; Dewailly et al. 1989; Dewailly et al. 1994; Hansen 1998; Van Oostdam et al. 2004). An early alarm signal of elevated polychlorinated biphenyl (PCB) levels in the Arctic environment published by Dewailly et al. in Dewailly et al. 1989 alerted Arctic governments to the presence of long-range transported contaminants in the Arctic. This led to the eight Arctic countries (Denmark, Iceland, Sweden, Norway, Finland, Russia, Canada and the USA) adopting the Arctic Environmental Protection Strategy and established the Arctic Monitoring and Assessment Programme (AMAP) to implement this strategy. AMAP was established as a working group under the Arctic Council (http://www.amap.no) in 1996 and has a circum-Arctic pollutant monitoring programme that includes more than 25 years of contaminant monitoring data in all Arctic environmental matrices. Because of their initiation and support of continuous monitoring and assessment activities, AMAP is today considered an important contributor to shaping the global and regional regulation of POPs within the European Union, under the UNEP Stockholm Convention on POPs and the Hemispheric Transport of Air Pollutants (HTAP) Aarhus Protocol, as well as by many national regulatory bodies. Based on the scientific evidence of their potential to pose a risk to human health and the environment in areas remote from sources, the production of legacy POPs and their usage is now globally regulated under the UNEP (Stockholm) Convention for the Protection of Human Health and the Environment from POPs. However, due to rapid developments and advancements in the technologies applied for pollutant analysis and toxicology, as well as ongoing risk assessments, new or ‘emerging’ organic contaminants are continuously identified and added to priority lists for international POPs monitoring (Fang et al. 2015; Magulova and Priceputu 2016).

## Scientific motivation

Arctic long-term monitoring of pollutants, including PCBs, dates back as far as 40 years for some environmental compartments and cover periods with less rapid environmental changes compared to today (Bonefeld-Jørgensen 2010; Hansen et al. 2002; Heidam et al. 2004; Hung et al. 2010). Changes in Arctic environmental conditions are now rapid and dynamic (Macdonald et al. 2003; Olsen et al. 2011; Parkinson and Berner 2009; Wöhrnschimmel et al. 2013). Therefore, changes in chemical distribution profiles, uptake rates and degradation pathways may serve as early warning indicators for direct and/or indirect effects of the currently observed Arctic environmental changes on the presence and impact of POPs in the Arctic. Knowing this, and having access to several long-term studies from large circum-Arctic studies, a group of international scientists led by AMAP undertook a comprehensive European research initiative under the European Union’s 7th Research and Innovation funding programme (FP7) entitled: ‘Arctic Health Risks: Impacts on health in the Arctic and Europe owing to climate-induced changes in contaminant cycling’ (*ArcRisk*)*.*

This review highlights the interdisciplinary research and key findings of *ArcRisk* on climate-induced changes of POP cycling in the Arctic environment using PCBs as a useful example. PCBs were included among the group of pollutants that was intensively studied in the *ArcRisk* project and are among the most well-investigated POPs worldwide (Olsson et al. 2010). Furthermore, PCBs are probably the best-understood POPs group in terms of physical-chemical properties, emissions, pathways and observed concentrations in the global environment (Beyer and Biziuk 2009; Carpenter 2006; Faroon and Ruiz 2015; Fernandez-Gonzalez et al. 2015; Henry 2015; Korrick and Sagiv 2008; Peakall 1972; Ross 2004; Safe 1994). Therefore, they were chosen as a case study performed within *ArcRisk* for evaluating the performance of environmental fate and distribution models and as a benchmark for other POP-like substances in a set of climate change scenarios. Even though PCBs are banned, they are still present in large quantities in urban environments as well as the environment and they will remain so for at least another century (Kallenborn et al. 2012). In addition, evidence for new PCB sources that have the potential to contribute to Arctic environmental pollution have been presented recently (Bogdal et al. 2014; Diamond et al. 2010; Gasic et al. 2010; Hu and Hornbuckle 2010; Pedersen et al. 2011; Vorkamp 2016). PCBs comprise of a total of 209 separate congeners and are therefore discussed in this review as total PCBs, as the sum of different (but environmentally abundant) congeners (mainly the ICES PCB_7_ congeners; PCB-28, 52, 99, 101, 118, 153 and 180) and as individual congeners, depending on available data. This paper is based on internal, unpublished reports from the ArcRisk project and on results from peer-reviewed papers within the project. The aim is to provide an overview of the research conducted within the *ArcRisk* project as a whole and combining results from all scientific areas including modelling, empirical investigations and meta-analysis of human health related to PCB. The present review begins with overviews of emissions of PCBs and their transport to the Arctic, followed by their environmental distribution and, finally, the impact that PCBs have on people living in the Arctic. Where possible, the aspect of climate change on the fate of PCBs in the Arctic is taken into account.

## Current PCB emissions and pathways to the Arctic

### Emission estimates

PCBs were used extensively during the 1950s to 1970s, mostly in industrial applications (such as coolants and insulating fluids) and as additives and sealants in building materials. After the 1970s, the production and use of PCBs were gradually restricted in many countries, and global emissions started to decrease. However, the decline in emissions lags strongly behind the rate of phase-out of production, because stocks of products in-use and materials continue to release PCBs to the atmosphere throughout their lifetime. Waste dumps, decommissioning sites as well as in-use stocks represent primary sources of today (Bogdal et al. 2014; Diamond et al. 2010; Gasic et al. 2010).

Furthermore, PCBs that have accumulated in the abiotic environment (sediment, water, soil, snow and ice) can be remobilized and thereafter re-emitted and thus may contribute to a slower declining rate of PCBs in the global atmosphere. Re-emissions from secondary sources will eventually become more important than primary sources in a global long-term perspective (Armitage et al. 2011; Stemmler and Lammel 2012). Recent review on fate and distribution of PCBs in the Arctic confirms these model-based results with empirical information (Hung et al. 2012; Kallenborn et al. 2012; Muir and de Wit 2010; Sobek and Gustafsson 2014; Villa et al. 2017; Vorkamp and Riget 2014).

**Fig. 1:**
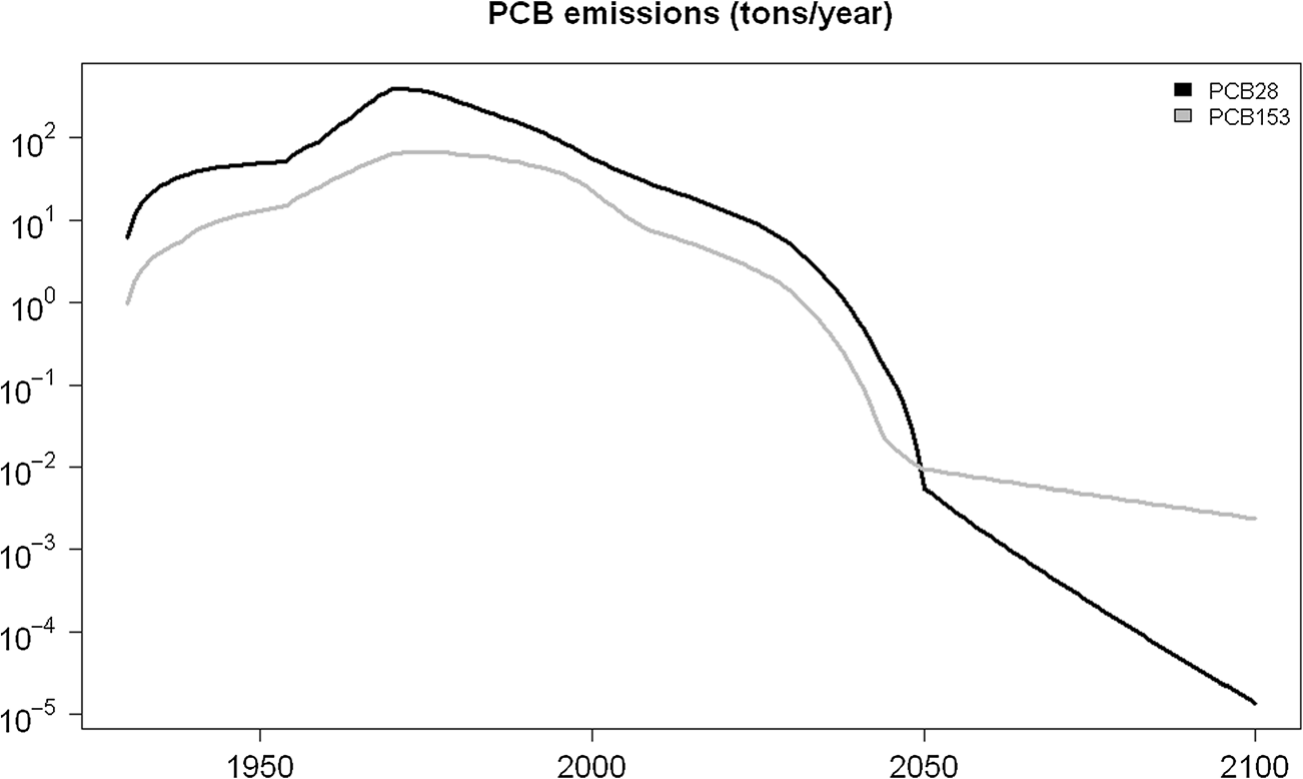
Global primary emissions scenarios of PCB28 and PCB153 to air (high-end estimate), according to Breivik et al. (2007)

In the modelling studies considered and applied in the *ArcRisk* project, the high-end emission scenarios for PCB28 and PCB153 estimated by Breivik et al. (Breivik et al. 2007) were used (Fig. 1), and the annual emission values were translated into monthly releases. It is important to note that the global emission inventory aimed at quantifying the ‘big picture’ in terms of global historical releases to air. The inventory may not accurately reflect actual emissions of a specific congener at a specific location or time. Local sources, still present within the Arctic, such as waste dumps, industrial installations and old settlements, may thus not be accurately represented in the global emission scenarios. Recent studies have shown the impact of these sources on the local environment on, e.g. Svalbard (Pedersen et al. 2011).

Nevertheless, atmospheric long-range transport is still considered to be a major route for the global environmental distribution of PCBs into remote areas. Even today, transport of contaminated air masses to the Arctic still occurs from regions in industrialised countries, where PCBs are still emitted from various sources (Hung et al. 2016). In spite of limited historical production and use of these chemicals, surprisingly high concentrations of PCBs were recently reported in some developing countries, partly attributed to transboundary export followed by poorly regulated recycling and disposal of relevant wastes (Bogdal et al. 2014; Breivik et al. 2011; Gasic et al. 2010; Hung et al. 2016; MacLeod et al. 2014).

### Spatial variations of PCB concentrations in the Arctic

An overview of atmospheric concentrations of ∑_7_PCBs at different sites in European and Arctic areas is available from www.genasis.cz and www.pops-gmp.org where additional information can be found (including other compound groups). Based on this comprehensive data, the highest atmospheric concentrations of PCB_7_ are present in central and eastern Europe where levels higher than 500 pg/m^3^ can occur and indicate the presence of ‘hot spots’ areas. The highest PCB levels at background sites were also found in central and eastern Europe (Halse et al. 2011). The concentrations in Arctic areas are generally in the range < 5–30 pg/m^3^ (Hung et al. 2010, 2016).

**Fig. 2:**
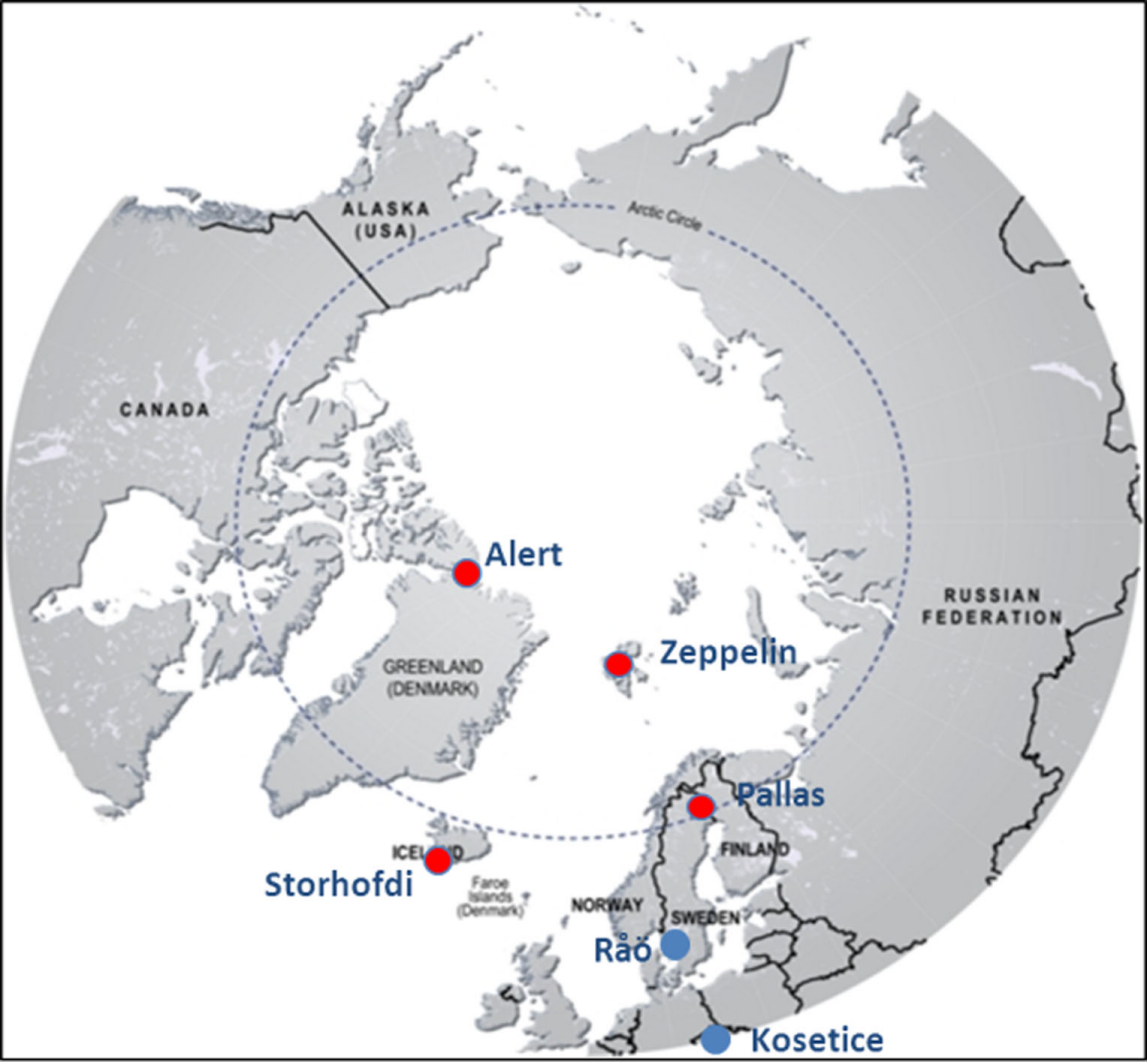
Long-term monitoring stations for PCBs and other air pollutants. Red dots indicate Arctic stations and blue dots indicate European stations included in ArcRisk. Pallas and Zeppelin were directly included and used in the ArcRisk project for sampling in the Arctic. Data from the other Arctic stations was incorporated, but no own sampling campaigns were launched there

Levels and long-term time trends of PCBs and other POPs are monitored on a continuous basis in the Arctic atmosphere within different national monitoring programmes. The Arctic sampling sites and the European reference sampling sites used within the *ArcRisk* project are shown in Fig. 2. The air monitoring at these stations has been carried out since the early 1990s and quality-controlled data are continuously reported to the AMAP and the European Monitoring and Evaluation Programme (EMEP) programmes (Hung et al. 2016; Tørseth et al. 2012).

The average PCB concentrations at the four major Arctic atmospheric monitoring sites of Alert (Canada), Pallas (Finland), Stórhöfði (Iceland) and Zeppelin (Svalbard/Norway) may vary due their proximity to sources and sources and their geographical location (latitude and longitude) but also due to environmental factors such as marine or continental influence, altitude and the prevailing meteorology for the respective stations (e.g. see Hung et al. 2010, 2016).

**Fig. 3:**
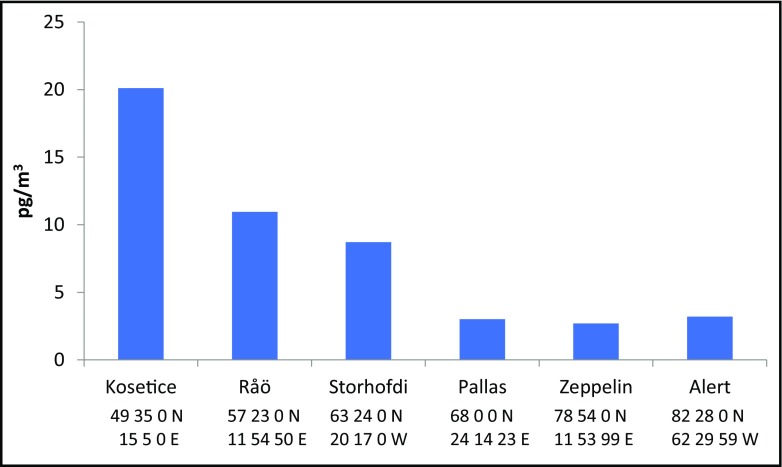
Yearly average atmospheric concentrations in 2009 of Σ_7_PCBs (28, 52, 101, 118, 138, 153, 180) (please note that PCB138 is not included at Alert)

The yearly average atmospheric concentrations of PCB_7_ at the high Arctic stations (Alert; northeast Canada and Zeppelin; Svalbard) and the European sub-Arctic stations (Stórhöfði; Iceland and Pallas; northern Finland 60–66° N) in 2009 are shown in Fig. 3, where also the PCB concentrations from background sites in central Europe (Košetice in the Czech Republic) and southern Scandinavia (Råö, at the Swedish west coast) are shown. An obvious decrease in the PCB concentrations from central Europe and southern Scandinavia to the Arctic areas is evident. The PCB concentrations at Stórhöfði were comparable to levels in southern Scandinavia (Råö) and the concentrations at Pallas were at the same level as those at the high Arctic stations (Hung et al. 2010, 2016).

During 2009, the concentrations of PCBs at the Arctic stations, Zeppelin (east) and Alert (west) were at the same level, in contrast to the period 1993 to 2006 for which Hung et al. (2016) reported a west–east gradient, the eastern stations being characterised by higher PCB levels.

### Time trends and seasonal cycling in atmospheric PCB patterns

Long-term trends and seasonal cycles of PCBs at different Arctic atmospheric monitoring stations may reveal the influence of regional, local and seasonal factors and are thus expected to give essential information to assess the effectiveness of control strategies. The *ArcRisk* work builds on extensive time series of POPs in the Arctic. Results from these time series are presented in this chapter, including results that have not been published elsewhere.

**Fig. 4:**
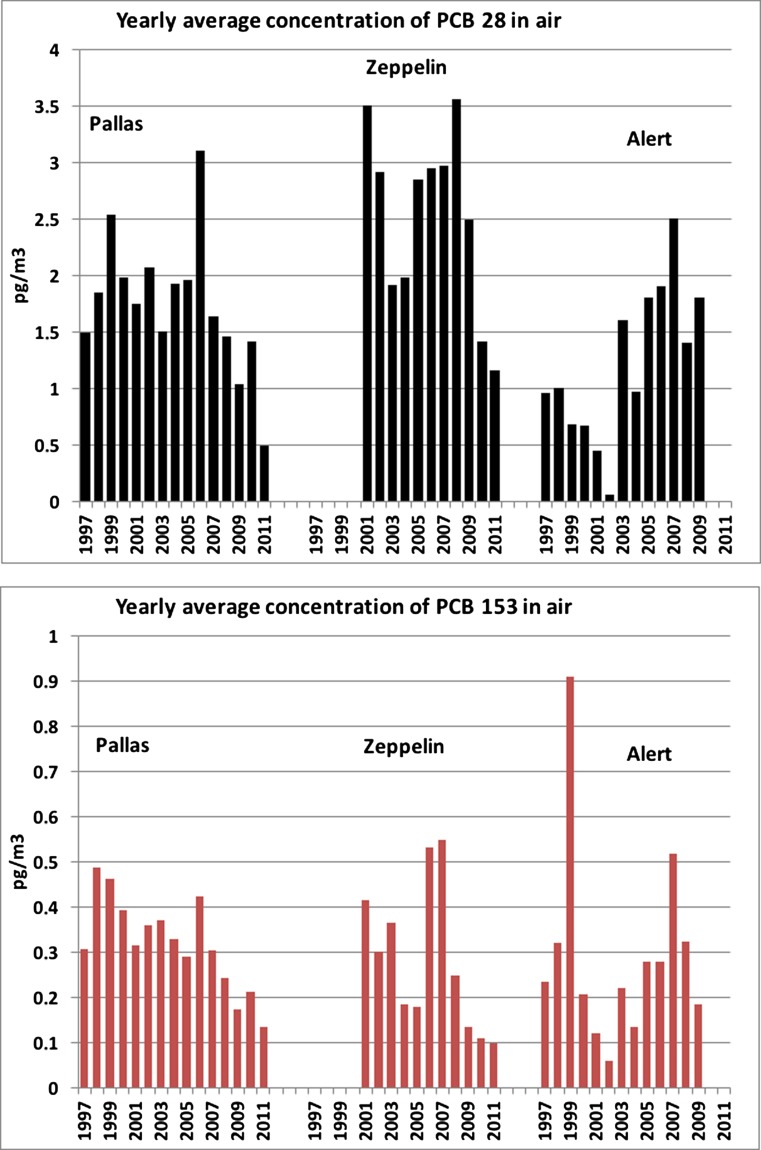
Yearly average atmospheric concentrations of PCB28 and PCB153 at Pallas, Alert and Zeppelin

The atmospheric PCB concentrations in the Arctic have shown a continuous decreasing trend over the past decades, after the international regulation of PCB production and usage was enforced (Hung et al. 2016). Hung et al. (2016) showed a general decline in the concentrations at Pallas, Alert and Zeppelin over the period 1998–2012. Declining PCB concentrations have also been identified at Pallas for the period 1996–2008 (about 3%/year for ∑_7_PCB), which was similar to the decline observed in southern Scandinavia earlier (Backe et al. 2002). The yearly average atmospheric concentrations of PCB28 (tri-CB) and PCB153 (hexa-CB) at Pallas, Alert and Zeppelin between 1997 and 2009 are shown in Fig. 4. Further details on more comprehensive Arctic atmospheric monitoring can be found elsewhere (AMAP 2016; Hung et al. 2016).

The concentrations of both PCB28 (tri-CB) and PCB153 (hexa-CB) were generally higher at Zeppelin in comparison to Pallas and Alert for several of the years, but the difference is levelling out. For the Zeppelin station, increasing trends for medium chlorinated (penta- to hexa-chlorinated CBs) were reported in the period 2004 to 2009 (decreasing PCB levels after 2009). Occasionally elevated levels during this period were associated with biomass burning events in Eastern Europe and boreal forest fires in North America, followed by transport of contaminated air into the Svalbard region, which may be seen in the context of a changing climate in the boreal region (Eckhardt et al. 2007; Kelly et al. 2013). Re-emission of (lighter) PCBs from oceans and snow caps might also contribute to increasing PCB concentrations in the Arctic atmosphere (Hung et al. 2016). Pallas showed a similar trend as Zeppelin, with increasing concentrations until 2006 followed by a decreasing trend thereafter. Modelling explained why the concentration of pollutants in the atmosphere above Svalbard correlates with the Arctic Oscillation, whereas this is not the case above Greenland (Octaviani et al. 2015). The Arctic Oscillation is a regular oscillation of the atmosphere above the Arctic that creates differences in atmospheric pressure. Pollutant flows from Europe, which correlate positively with the Arctic Oscillation, maintain the concentrations above Svalbard. The pollutant concentrations above Greenland, however, are determined by flows in the Canadian Archipelago, where air currents are in a reverse relation with this oscillation.

### Atmospheric deposition pathways

**Fig. 5:**
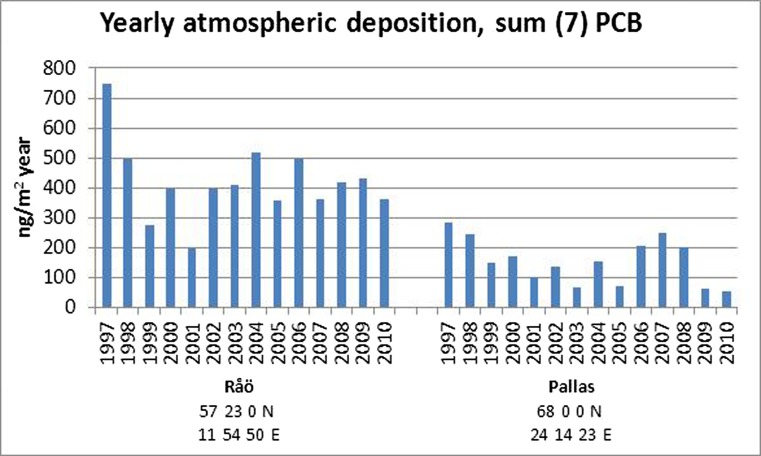
Yearly atmospheric deposition fluxes of Σ_7_PCBs (28, 52, 101, 118, 138, 153, 180) at Råö and Pallas (bulk deposition) as reported in the final *ArcRisk* report (www.arcrisk.eu)

Deposition from the atmosphere (both wet and dry deposition) is the dominant process for the input of PCBs into both terrestrial and marine Arctic environments (Garmash et al. 2013; Kallenborn et al. 2007; Malmquist et al. 2003). At Pallas, long-term deposition measurements of PCBs have been carried out since 1997. The results from these measurements are shown in Fig. 5 where the deposition fluxes from Råö at the Swedish west coast are included as comparison. Deposition data from long-term monitoring in the high Arctic are not available.

The annual average deposition fluxes of the Σ_7_PCBs at Pallas ranged from 100 to 300 ng/m^2^/year in the period 1997–2010. The deposition fluxes at Pallas are about twofold lower than those measured in southern Scandinavia. The highest deposition fluxes occurred during the first measurement years and the lowest, like the air concentrations, during recent years. However, unlike for PCB concentrations in air, no decreasing trend was observed (Fig. 5).

As the PCB levels in air at Pallas and Zeppelin (Hung et al. 2016) are reported in the same order of magnitude, we could assume that yearly deposition at Zeppelin should be considered in the same range as for Pallas. However, total atmospheric deposition fluxes are dependent not only on gaseous and particulate phase concentrations but also on precipitation type and rate, ambient temperature and atmospheric particulate matter concentration and mass size distribution (see also discussion on PCBs in snowpack in section ‘Emission estimates’). Wet deposition at colder temperatures (e.g. close to 0 °C) is more efficient at scavenging semi-volatile organic chemicals such as PCBs from the atmosphere, compared to wet deposition at warmer temperatures (see Lei and Wania (2004)). The dominance of snowfall in annual precipitation at more northerly sites may account for the lack of a decline in observed deposition fluxes (Hansen et al. 2006; Hansson et al. 2006).

## Important inter-compartmental transfer processes in the Arctic

### Processes and pathways in Arctic snow and ice

The extensive usage history of PCBs (documented since the 1920s; Kimbrough and Jensen (1989)) until their global ban during the 1980s/1990s has resulted in their ubiquitous global distribution. Atmospheric and chemical processes drive transfer between environmental compartments in the Earth system including transfer to the Arctic, with these processes continuing long after PCBs have been phased out from usage. Atmospheric deposition to the extensive sub-Arctic catchment areas of the larger Arctic-draining rivers has provided considerable PCB loads to the rivers. In combination with point sources of PCBs located within these catchment areas, the rivers serve as important sources of PCBs to the Arctic regional seas (Carrizo and Gustafsson 2011b; Carroll et al. 2008; Rawn et al. 2001; Sobek and Gustafsson 2014). Atmospheric transport and deposition via snow fall have been identified as important transfer process in the Arctic including PCB deposition fluxes (Garmash et al. 2013; Hansen et al. 2006; Pavlova et al. 2014). Rapid redistribution processes during surface snow weathering determine whether the respective contaminant is re-evaporated, released into the soil or retained in the snowpack throughout the season (Herbert et al. 2005). However, the contribution of PCBs from melting snow and sea ice to the total PCB content in the Arctic marine environment is low compared to input from Arctic rivers. Nevertheless, rapid thawing processes and the changing Arctic marine cryosphere in a warmer Arctic could impact PCB exposure to ice-associated algae and fauna during the spring algal bloom (Carroll et al. 2008). However, deposition of PCBs with snowfall and subsequent accumulation in the seasonal snowpack, as well as accumulation in sea ice and colder Arctic waters, are still not sufficiently understood and quantified (Gustafsson et al. 2005; Hansen et al. 2006; Herbert et al. 2005).

Deposition processes and snow ice interactions of PCBs have been a research focus in the Canadian Arctic for many decades (Macdonal et al. 1996). A series of studies have been conducted to shed light on PCB accumulation and deposition in the Canadian Arctic. A winter field campaign in the Canadian Arctic measured PCBs in the surface snowpack from April through to early June 2008, just prior to ice breakup as part of a larger campaign to look at contaminant and nutrient flows associated with ice floes and associated ice leads (Pucko et al. 2015; Grannas et al. 2013). The mean concentration of ∑_29_PCB in snow was 256 ± 177 pg/L, although two fresh snowfall layers sampled in May displayed higher concentrations of 545 and 611 pg/L (Codling 2012). Excluding these two events, the average concentration in the snowpack was 185 ± 85 pg/L, which is substantially higher than PCB concentrations in surface seawater, indicating the efficiency of the snowpack to scavenge and accumulate semi-volatile organic chemicals from the overlying atmosphere. The PCB concentrations in the winter marine snowpack were lower than PCB concentrations measured previously in surface snow layers in northern Norway by a factor of ~ 2–5 (Herbert et al. 2005) but markedly higher (by 27-fold) than concentrations previously measured in ice-rafted snow collected in the marginal ice zone of the Barents Sea (Gustafsson et al. 2005) and distinctly higher than in Antarctica (Desideri et al. 1994; Vecchiato et al. 2015). The Barents Sea study was conducted during a Swedish research expedition in the marginal ice zone during July 2001, when the snow had already undergone substantial metamorphosis associated with repeated freeze-thaw cycles resulting in the likely loss of gaseous PCBs, either through volatilisation or re-partitioning to particulate matter. Interestingly, in the marginal ice zone, the particle-bound concentration was 99 pg/L (based on ∑_15_PCB measured in a composite snow sample marked by high levels of particle organic carbon (668 ± 50 mg POC/g)), while a mean concentration of 14.6 ± 11.9 pg/L (∑_15_PCB) of the particle-bound PCBs was measured. Thus, this earlier report indicates substantial re-processing and loss of PCBs during ageing and partial melt of the marine snowpack.

**Fig. 6:**
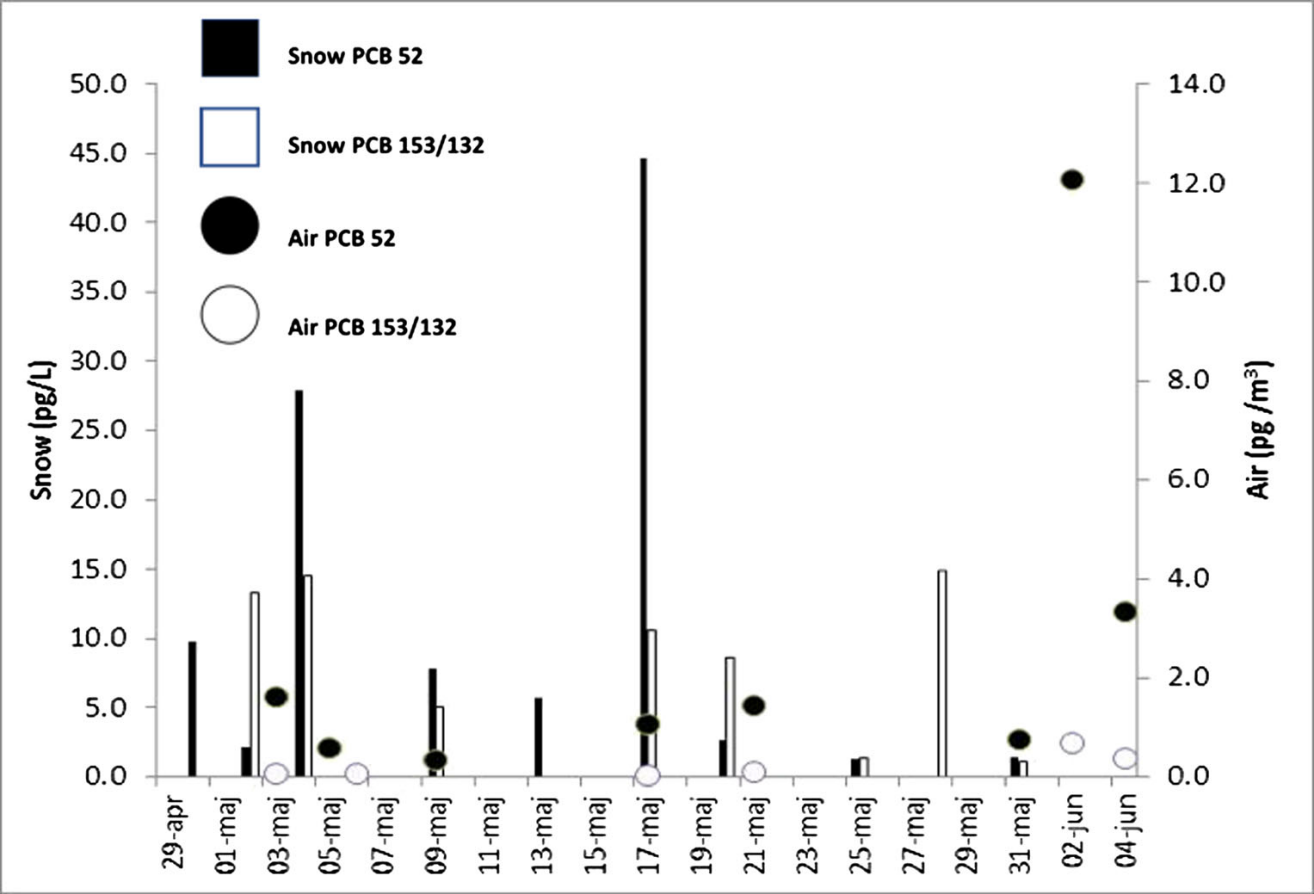
Concentrations of PCB52 and PCB153/PCB132 in the ice-rafted snowpack of the Beaufort Sea (Arctic Canada) during the late winter season (April–June 2008) (Codling 2012)

During the more recent campaign in the Tromsø (North Norway) area, the vapour-sorbed PCB concentrations in the snowpack accounted for approximately 80% of the PCB burden with ~ 20% associated with particles. Figure 6 illustrates the spring time series of PCB52 (tetra-chlorinated PCB) and PCB153/132 (hexa-chlorinated PCBs) in the marine snowpack in Beaufort Sea. Aside from the fresh snowfall event on 17 May, PCB52 concentrations in snow (white bars) declined over the time series notably once air temperatures started to exceed 0 °C (for part of each 24-h period). The heavier PCB153/132 does not show this trend (Brorström-Lundén et al. 2013). Losses of the lighter PCBs and, hence, enrichment of the heavier PCBs are due to volatilisation losses as the snowpack ages and can be attributed to changes in snow structure (specifically loss of snow surface area as the snow ages) (Stocker et al. 2007). To assess the role of the snowpack and first-year sea ice in supplying accumulated contaminants to the polar mixed layer of the Beaufort Sea, a late season snowpack and ice column inventory was calculated. The PCB congeners (AMAP ‘10’; PCB-28, 31, 52, 101, 105, 118, 138, 153, 156 and 180) were selected based on the chemical concentrations measured in the snowpack and sea ice, assuming full ice cover (and hence an ice-rafted snowpack) over the entire Beaufort Sea, prior to ice breakup in June. The ∑_10_PCB burden in both snow and ice is presented in Table 1 and was estimated as 6.17 ± 3.34 kg (Codling 2012). This value is similar to a recent estimate of the PCB inventory for the polar mixed surface layer of the Beaufort Sea of 4.47 kg (Carrizo and Gustafsson 2011a). However, in that study. a much smaller area of the Beaufort Sea was selected for the calculations (178,000 km^2^ and 124 m depth). When this area is used for ice cover instead, the PCB inventory for snow and ice becomes ~ 2.5 kg. Assuming minimal ice export from the Beaufort Sea, then the release of PCBs from the snow/ice system into surface seawater (i.e. the polar mixed layer ~ 40 m depth) during final melt would yield concentrations in seawater of ~ 0.16–0.53 pg/L or ≈ 5–18% of the PCB concentration present in surface waters of the Beaufort Sea. These values provide a first quantitative estimate for the role of the sea ice system in storing and releasing POPs to seawater along a seasonal temperature and cryosphere extension pattern (Carrizo and Gustafsson 2011b).

**Table 1:** Estimated load (kg) of PCBs in the sea ice system of the entire Beaufort Sea prior to ice breakup

	Snowpack	Ice
PCB18	0.45 ± 0.21	2.66 ± 1.33
PCB31/28	1.87 ± 0.96	0.25 ± 0.13
PCB52	0.13 ± 0.05	0.07 ± 0.03
PCB99/101	0.29 ± 0.20	0.01 ± 0.007
PCB118	0.03 ± 0.04	NR
PCB153/132	0.22 ± 0.16	0.02 ± 0.01
PCB138	0.17 ± 0.22	NR
Σ_7_PCB	3.16 ± 1.84	3.01 ± 1.50

Data from Codling (2012)

*NR* not reported

### Riverine transport as an Arctic distribution pathway

**Fig. 7:**
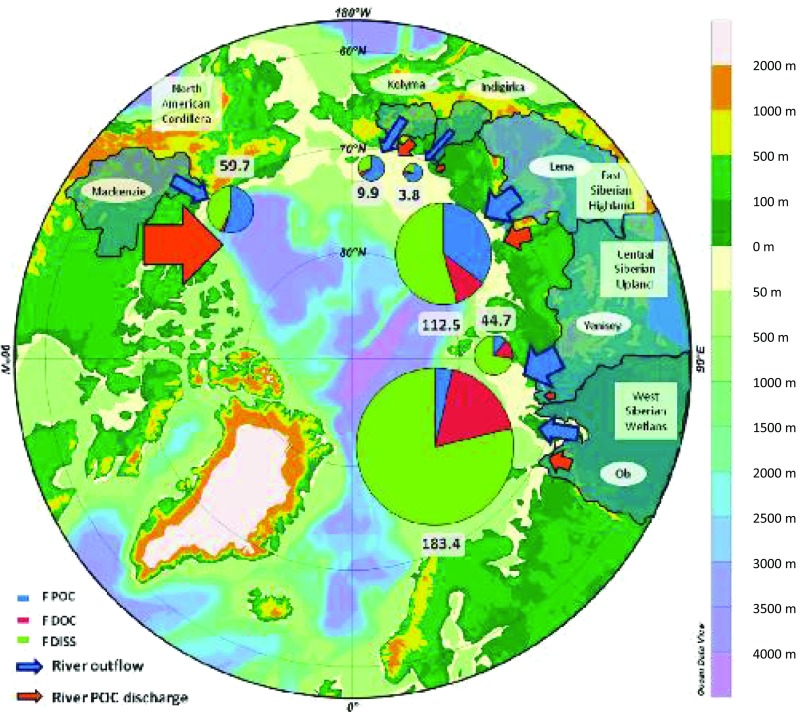
Σ_13_PCB fluxes (kg/year) estimated for the six major Arctic rivers are given for the dissolved (F DISS), DOC-associated (F DOC) and particulate-associated (F POC) fractions. Please note the figure is reproduced from Carrizo and Gustafsson (2011b)

The Arctic Ocean receives PCBs via deposition from the atmosphere, from drainage of the major Arctic-flowing rivers and through surface ocean currents entering the Arctic from the Atlantic and Pacific Oceans (Fig. 7). Pan-Arctic riverine fluxes of PCBs have been estimated based on recent ship-based campaigns that measured PCBs in the fluvial surface sediments in the estuaries of the six major Arctic-draining rivers (Ob, Yenisey, Lena, Indigirka, Kolyma and Mackenzie) and are currently seen as important PCB distribution pathways (Carrizo and Gustafsson 2011b; Carroll et al. 2008). Combined, these six rivers contribute on average 1935 km^3^/year of freshwater discharge to the coastal seas of the Arctic Ocean, the largest proportion of freshwater flows to the Arctic Ocean. The Σ_13_PCB fluxes (kg/year) are presented in Fig. 7. The highest PCB fluxes occurred for the two major Russian rivers, Ob and Lena—the rivers with the highest water discharge rates and with catchments that extend far to the south beyond the Arctic. The Σ_13_PCB fluxes from these rivers were estimated to be 183 kg/year for Ob and 113 kg/year for Lena. As comparison, the Mackenzie and Yenisey rivers have Σ_13_PCB fluxes of 60 and 45 kg/year, respectively, followed by the eastern Siberian rivers of the Kolyma and Indigirka, with fluxes of 10 and 3.9 kg/year, respectively. These fluxes are based on estimates made a decade ago, and with continuing climate-induced changes to the Arctic environment, it is likely that these fluxes will have changed. However, across these six rivers, the PCB congener composition differed, with the Russian rivers possessing relatively higher fractions of the penta- and hexa-chlorinated PCB congeners and the Mackenzie River (Canada) possessing higher fractions of the tri- and tetra-PCB congeners. This difference between the Russian and Canadian rivers is probably related to the major Russian technical PCB formulation of ‘Sovol’, which is composed of ~ 50% penta-chlorinated congeners. In order to complement the riverine flux assessment of PCBs, a recent evaluation of PCB concentrations in the Arctic coastal seas has been undertaken (Carrizo and Gustafsson 2011a). This assessment included an examination of the PCB congener composition to understand the influence of different source regions on the Arctic and the mode of transport, e.g. atmospheric vs. transport in water.

**Fig. 8:**
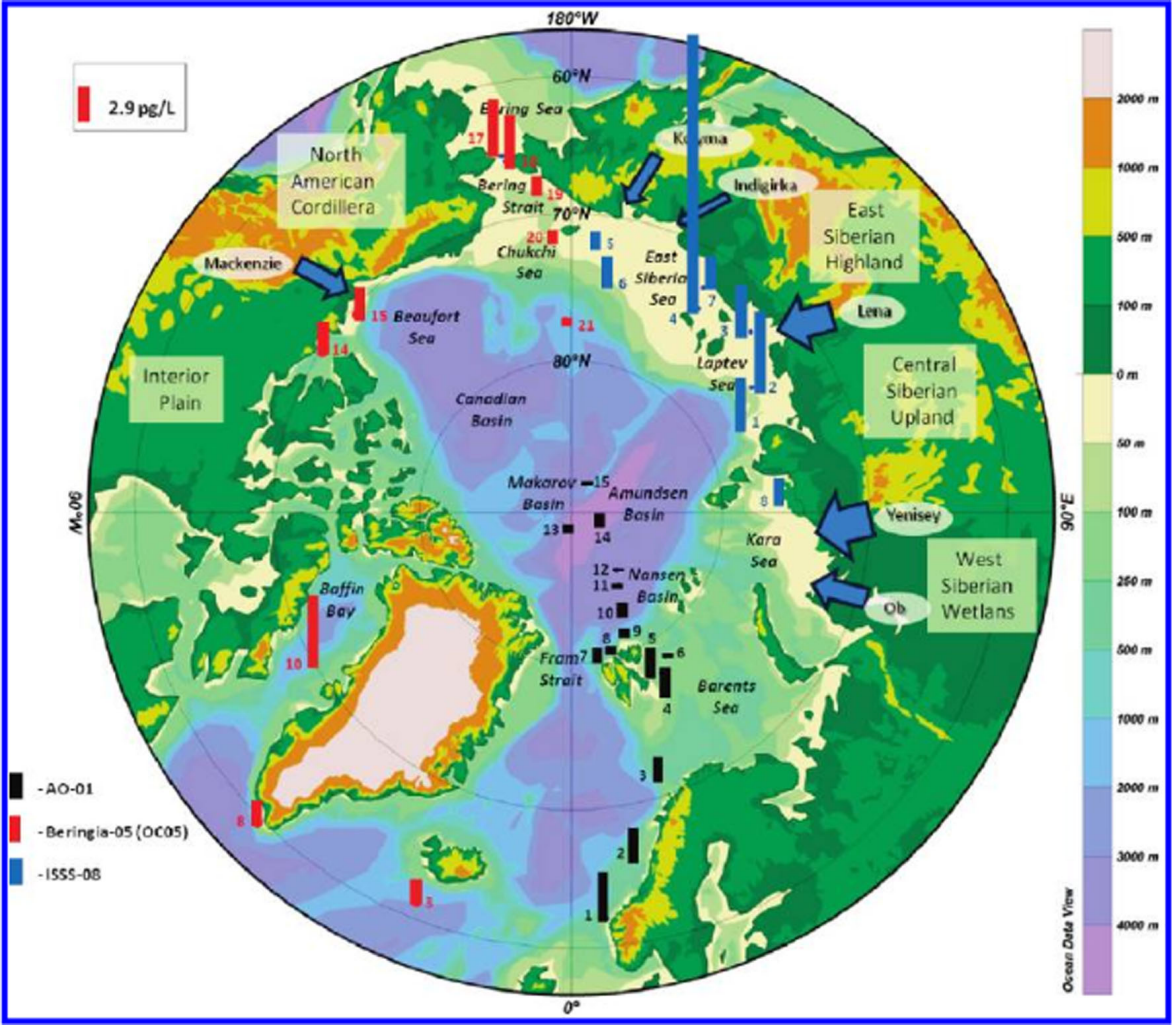
Σ_13_PCB concentrations (pg/L) (dissolved and particle-bound) in the surface waters (polar mixed layer) of the Arctic Ocean. The colour key indicates the research campaign; the numbers on the bars are station numbers. The concentration key is at the upper left. Please note the figure is reproduced from Carrizo and Gustafsson (2011b)

Figure 8 illustrates the spatial distribution of surface seawater PCB concentrations for the different geographical regions. Contemporary PCB concentrations across the Arctic Ocean range from (Σ_13_PCB) 0.13 to 21 pg/L, with higher concentrations in the shelf seas than in the central Arctic Ocean. Tri-chlorinated PCBs contribute about 50% of the total PCB loading in the surface waters of the eastern Arctic (Bering, Chukchi and Beaufort seas), suggesting a predominantly atmospheric source, whereas the hexa-chlorinated PCBs are more abundant in the western part of the Arctic (Barents and Greenland seas), suggesting the influence of waterborne transport from regions with previous heavy PCB usage such as northern Europe and North America.

The first Pan-Arctic assessment by Carrizo and Gustafsson provided a comprehensive overview of PCBs in marine surface waters, including a baseline to model the uptake of PCBs into the marine food webs and also the basis to forecast future changes in PCB exposure for different regions of the Arctic (Carrizo and Gustafsson 2011a, b).

### Arctic soils

The terrestrial environment has been shown to be of importance for global POP cycling, and soils and forests in the northern hemisphere are recognised as storage compartments with a large capacity for POPs such as PCBs (Meijer et al. 2003; Kallenborn et al. 2012). We applied a global modelling using a multi-compartment chemistry-transport model for the decade 2001–2010 (Stemmler and Lammel 2012). This model forecast that high-chlorinated PCBs such as PCB153 will increase with 0.6%/year in Arctic soils, despite decreasing primary emissions (since the 1970s) and unlike in other regions. This feature was not shown for the low-chlorinated congeners. In comparison to mid-latitudes, the low-medium chlorinated congeners are enriched in the polar atmosphere as well as in ground compartments. These results indicate the strong significance of secondary sources for the cycling and subsequent accumulation of these contaminants in polar ecosystems.

## PCB occurrence in biota and trends in the Arctic

PCBs have been measured in a number of biological matrices in the Arctic environment. A west-to-east gradient for PCB levels has been identified, with the highest levels occurring in the Eastern Arctic (Hobbs et al. 2003; Muir et al. 1992; Norstrom et al. 1998; Verreault et al. 2005). In particular, Arctic top-predator animals have accumulated considerable PCB burdens in their lipid tissues (Bjerregaard-Olesen et al. 2017; Bustnes et al. 2017; Dallaire et al. 2013; Nost et al. 2017; Pedro et al. 2017a; Ryan et al. 2013); AMAP 2016). During the past decade, several review papers have summarised information about the distribution patterns and ecotoxicology of PCBs in the Arctic (Derocher et al. 2003; Fisk et al. 2005; Letcher et al. 2010; Sagerup et al. 2009; Tartu et al. 2014; Tartu et al. 2015; Toft 2014; Verreault et al. 2006; Vijayan et al. 2006).

As documented in national and circum-Arctic monitoring (Hung et al. 2016; Letcher et al. 2010; Muir and de Wit 2010; Olsen et al. 2011), PCB concentrations in many environmental compartments, including biota, have continuously decreased over recent decades. Riget et al. (2010) studied time trends of PCBs in Arctic biota: fish, seabirds, marine mammals and reindeer. They found a decrease in the annual mean concentrations per year of 1.2 and 1.9% for PCB153 and ∑_10_PCB, respectively. The authors used 40 (PCB153) and 16 (∑_10_PCB) time series covering at least 6 years for samples collected in Canada, Iceland, Greenland, Norway and Sweden. Around 40% of those time series showed statistically significant decreasing trends across the Arctic area. However, in a few cases, a statistically significant increase in concentrations was seen (blue mussels, Iceland; freshwater fish, Canada; marine mammal population, Faroe Islands) in those trend studies (AMAP 2016 (Riget et al. 2010).

A short overview of PCB distribution in biota and its consequences in the Arctic environment, based on the work conducted within *ArcRisk*, is presented here.

### Levels in Arctic biota

A variety of earlier trend studies are reporting data from North Atlantic cod (*Gadus morhua*) in the Arctic (Ballschmiter and Zell 1980; Cleemann et al. 2000; Foreid et al. 2000; Haukas et al. 2007; Hellou et al. 1993; Pedro et al. 2017b; Sturludottir et al. 2014). Letcher et al. (2010) reviewed ∑PCB concentrations in mammals in the Arctic and found that lipid-normalised concentrations (lw) in tissues from whale species varied between 451 and 230,000 ng/g lw. In ringed seal, the mean PCB concentrations in blubber ranged between 200 and 1370 ng/g lw and the blood concentrations in Stellar sea lions between 3692 and 18,000 ng/g lw. Polar bears had PCB concentrations in fat varying between 1138 and 9100 ng/g lw. Long-term time series of PCBs in Arctic marine mammals are, in general, decreasing, although there are exceptions that can be linked to changes in diet or changes in environmental processes that impact run-off and re-emissions (McKinney et al. 2011); AMAP 2016).

In the ArcRisk project, several Arctic food products from Nuuk, Greenland were analysed for PCBs and other POPs (Carlsson et al. 2014a). ArcRisk results showed that food products derived from marine mammal species are contaminated with a variety of organic contaminants, such as a suite of perfluorinated alkylated substances (PFAS) as well as conventional legacy POPs that have been included in the Stockholm Convention for over a decade, such as PCBs. Not surprisingly, the highest PCB concentrations were found in narwhal mattak (skin and blubber), with a median concentration of 1147 ng/g lw. As a comparison, median concentrations of ∑PCB in seal meat and salmon were 302 and 227 ng/g lw, respectively. All samples were collected in the local food market in Nuuk, Greenland. The congeners PCB153 and − 138 were dominant (30% of the total PCB concentrations) in all samples investigated and even contributed 52% of the PCB load in the seal meat. PCB153, PCB138, PCB118 and PCB101 together contributed more than 50% of ΣPCB in all samples. The relative contributions of PCB118 and PCB153 were slightly higher in fresh salmon compared to smoked salmon, while the relative contributions of PCB101 and PCB149 were slightly higher in the smoked salmon. This indicates some influence of food processing on PCB profiles, although other congeners with a large relative contribution did not differ much (Carlsson et al. 2014a). The PCB levels (ng/g lw) in Greenlandic fish products found in this study are comparable to levels found in other studies from Arctic areas and to PCB levels in fish from European sites. As a follow-up to this study, a current report on shrimp and Northern halibut filet from Northern Norway confirmed the still prominent role of PCBs in the contaminant profile of marine species commercially exploited as seafood in the North (Carlsson et al. 2016).

The levels of ΣPCB in the smoked halibut from the Arctic were in line with levels in halibut from Tromsø (Carlsson et al. 2016) and Greenland (Johansen et al. 2004). However, the levels of PCBs in fish depend on factors such as trophic level, age and lipid content, as well as geographical distribution and the related exposure. Cod, which is a lean fish, will have higher levels of PCBs on a lipid-weight basis than fatty fishes like salmon. As an indication, a comparison of average PCB levels between cod and salmon on a wet weight basis shows lower levels in cod (3.96 ng/g ww) than in salmon (8–17.9 ng/g ww) even though cod feeds at a higher trophic level than salmon (Johansen et al. 2004). PCB concentrations in low trophic level biota (i.e. amphipods) have been studied and reported earlier from the Barents Sea (Evenset et al. 2016; Hallanger et al. 2011a). ΣPCB_7_ concentrations varied between 0.4 and 3.2 ng/g lw and are currently considered as background concentrations in Arctic zooplankton associated with the marginal ice zone. These studies also showed clear seasonal POP distribution differences that depend on environmental factors such as ice cover/melting as well as biological factors, e.g. feeding behaviour (Evenset et al. 2016; Hallanger et al. 2011b). A comparative study, in which enantiomer-selective distribution patterns of chlorinated pesticides in low trophic level organisms were associated with ocean current profiles in coastal Svalbard, indicates the influence of oceanographic and climate variables on the pollutant pathways (Carlsson et al. 2014b; Hallanger et al. 2011a). It is, thus, scientifically confirmed that even low trophic level organisms bioaccumulate organochlorine contaminants and supports earlier observations (Borga et al. 2005a, b). The transfer of legacy POPs including PCBs from the lower trophic level organisms into the top predators of the Arctic (i.e. polar bear, glaucous gull, polar fox) along a typical marine and/or terrestrial food web is usually associated with the transfer of lipids (Fisk et al. 2001a; Kleivane et al. 2000). The studies conducted within ArcRisk showed the importance of understanding how secondary sources may impact the environmental fate of PCBs in the food web in the light of a changing climate. Increased melting and run-off from land will have impacts on the PCB input and transfer through the food web, beginning at lower trophic levels and continuing through the food web up to humans as end consumers.

## Contaminant profiles in a changing Arctic climate

### Model-based forecasts of climate change impacts on PCB transport

Climate change is expected to significantly influence the global transport pathways and fate of persistent organic pollutants (Armitage et al. 2011; Bustnes et al. 2010; Dudley et al. 2015; Friedman et al. 2014; Kallenborn et al. 2012; Kraemer et al. 2005; Macdonald et al. 2005; Octaviani et al. 2015; Wöhrnschimmel et al. 2013). For PCBs, the forecast increase in temperature will enhance degradation of PCBs and increase volatilisation and hence mobilisation from primary sources and environmental surface media, such as seawater, ice and soils (Ma et al. 2011). Changes in precipitation patterns are expected to affect the transfer processes between air and surface (Kallenborn et al. 2012). Melting land and sea ice will reduce the non-biological available storage capacity and influence air-surface transfer. Finally, changes in oceanic and atmospheric circulation will lead to altered transport pathways of PCBs. However, the quantitative impact of these processes is associated with considerable uncertainties. Therefore, a comprehensive modelling exercise was conducted in the frame of the *ArcRisk* project whereby a variety of model approaches was chosen to examine the influence of climate change scenarios on PCBs. The modelling expert group applied the following tools: *Berkeley-Trent global contaminant fate model* (BETR Research), *Max-Planck Institute*—*Multi-Compartmental Chemical Transport Model* (MPI-MCTM), *Danish Eulerian Hemispheric Model* (DEHM) and *coupled atmosphere-ocean general circulation model* (ECHAM5-MPIOM). ECHAM5-MPIOM also served as input to some of the other models. Each of these models has been applied individually to assess and evaluate potential impacts of climate change on PCBs and other POPs in the Arctic. Specific results from each model are summarised below. Further details regarding parameters and sources for the models can be found in their respective sections below.

#### BETR Research

**Fig. 9:**
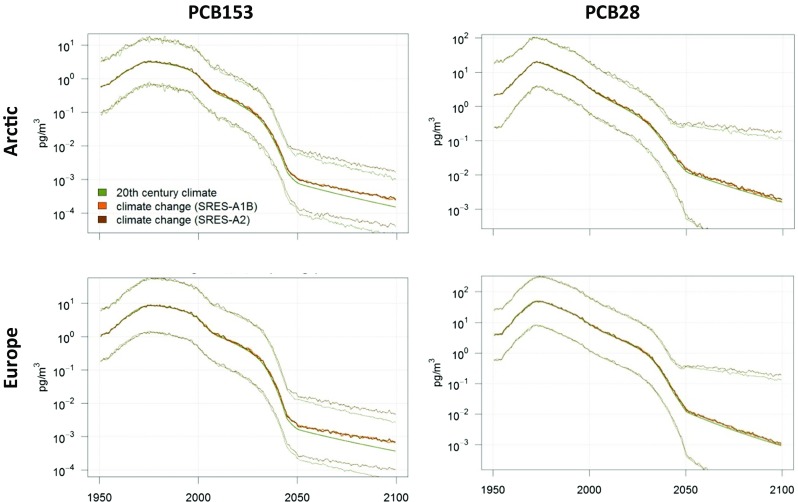
Modelled PCB153 (left column) and PCB28 (right column) concentrations in the Arctic (upper row) and European (lower row) atmosphere, with and without climate change. The green middle line represents no climate change while the brown line indicates what happens with PCB when climate change is taken into account. Uncertainties (95% confidence interval) are indicated for PCB153 with the SRES-A2 scenario by the thinner lines. Please note the figure is reproduced from Wöhrnschimmel et al. (2013)

**Fig. 10:**
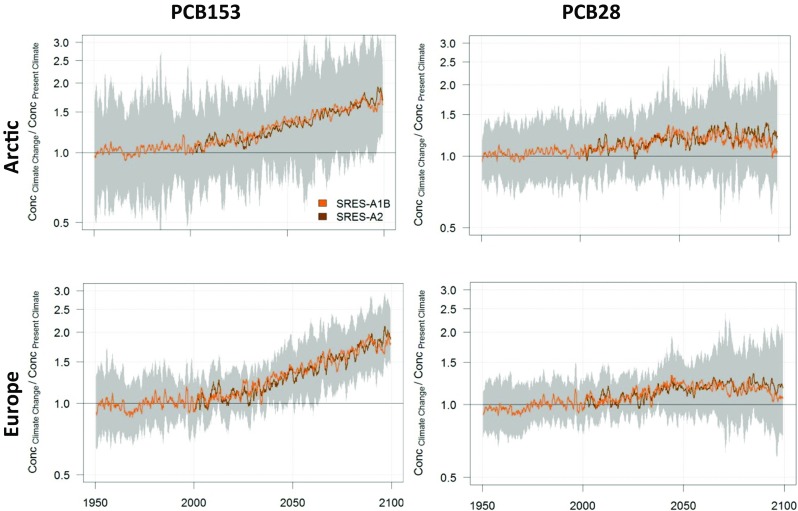
Modelled relative change of PCB153 (left column) and PCB28 (right column) concentrations in the Arctic (upper row) and European (lower row) atmosphere. The green middle line represents no climate change while the brown line indicates what happens with PCB when climate change is taken into account. Uncertainties (95% confidence interval) are indicated for PCB153 with the SRES-A2 scenario by the shaded area. Please note the figure is reproduced from Wöhrnschimmel et al. (2013)

**Fig. 11:**
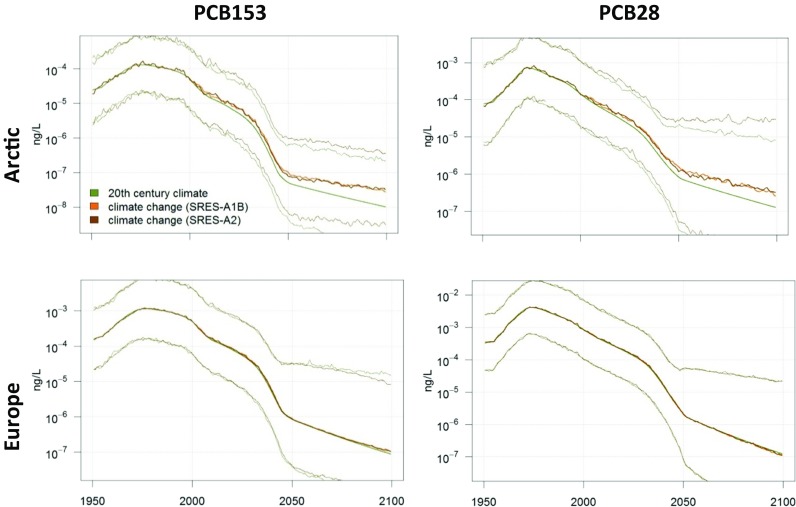
Modelled PCB153 (left column) and PCB28 (right column) concentrations in Arctic (upper row) and European (lower row) seawater, with and without climate change. The green middle line represents no climate change while the brown lines indicate what happens with PCB when climate change is taken into account. Uncertainties (95% confidence interval) are indicated for PCB153 with the SRES-A2 scenario by the thinner lines. Please note the figure is reproduced from Wöhrnschimmel et al. (2013)

**Fig. 12:**
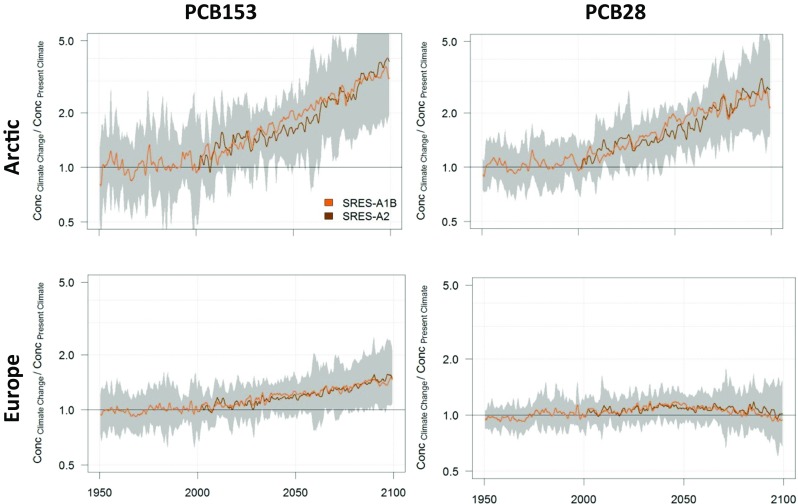
Modelled relative increase of PCB153 (left column) and PCB28 (right column) concentrations in the Arctic (upper row) and European (lower row) sea water. The green middle line represents no climate change while the brown lines indicate what happens with PCB when climate change is taken into account. Uncertainties (95% confidence interval) are indicated for PCB153 with the SRES-A2 scenario by the shaded area. Please note the figure is reproduced from Wöhrnschimmel et al. (2013)

**Fig. 13:**
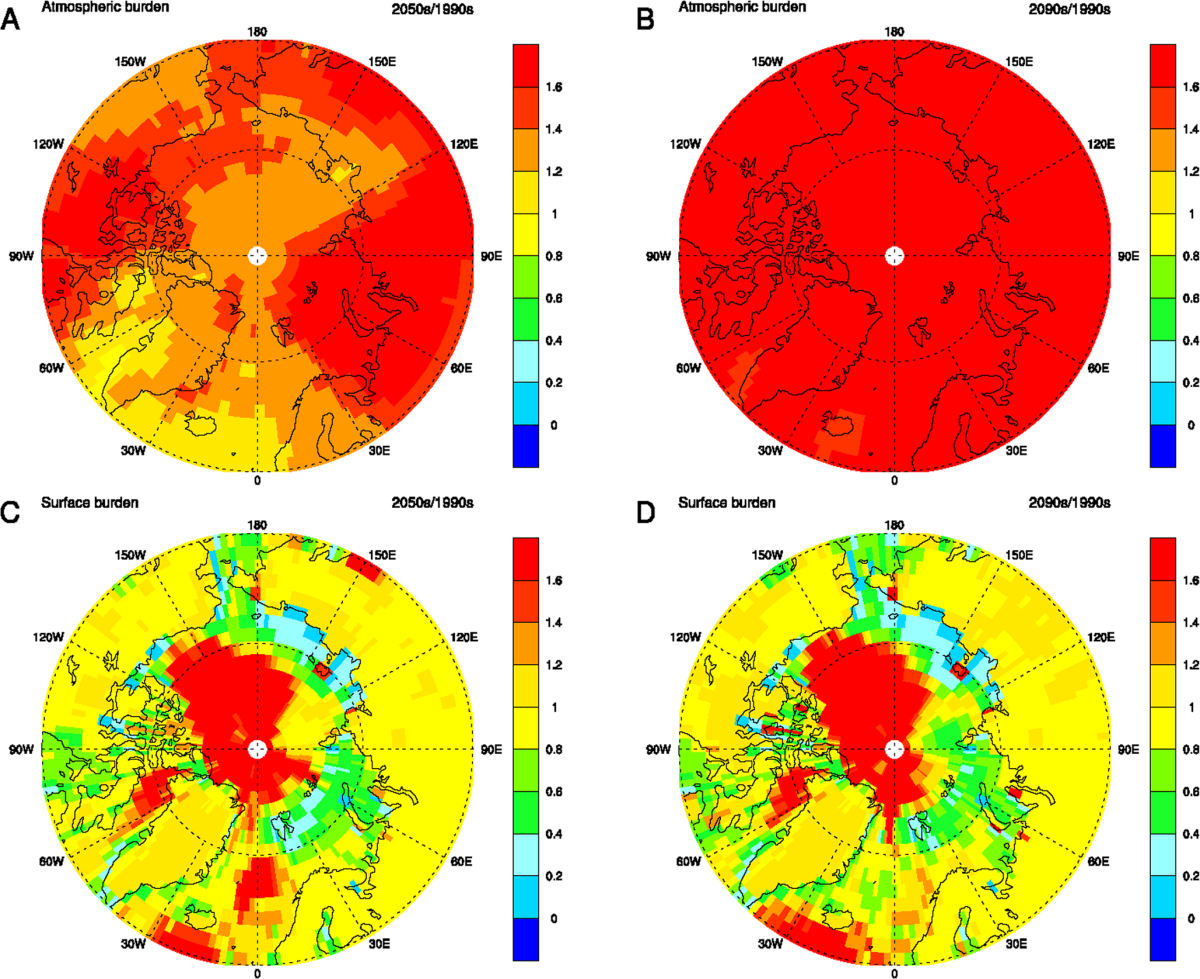
Modelled mapped ratio of concentrations of PCB153 in the winter (DJF) atmosphere (**a** and **b**, above) and surface compartments (**c** and **d**, below), with and without climate change (A1B scenario of the IPCC AR4) for the middle (**a** and **c**, 2050s/1990s) and end (**b** and **d**, 2090s/1990s) of the century. Values exceeding 1 indicate enhancement by climate change

BETR Research multimedia contaminant fate model was applied to model the impact of climate change on concentrations and distribution of PCBs in the Arctic. The emission history of PCB28 and − 153, which has been published earlier (Breivik et al. 2007), was used for this purpose. Environmental parameters were mainly built on the BETR Global defaults (MacLeod et al. 2011) and the ECHAM5/MPI-OM model outputs for different IPCC AR4 scenarios (Winton 2006). The chemical properties of PCBs that were used are described in (Lamon et al. 2009). Long-term monthly averages from 1980 to 2000 were used for present-day scenarios, while time-evolving fields were used (Figs. 9, 10, 11, 12 and 13) for the climate change scenarios.

Climate change is projected to have a larger impact in the Arctic on PCB153 in seawater compared to atmospheric PCB153. While the relative increase of PCB153 is projected to be a factor 1.5 in Europe, it is up to a factor of 3–4 in the Arctic. These increases are a result of the higher relative atmospheric concentrations (a factor of 1.5 higher concentrations in the Arctic and a factor of 2 higher in European atmosphere) in combination with increased deposition into the Arctic Ocean, which is also facilitated by the decreasing sea ice cover. PCB28 also shows a relative increase in the model (a factor of about 2.5–3) in the Arctic Ocean, while it is projected to decrease in European seawater. However, that decrease might be within the parameter uncertainties (Figs. 10 and 12) and so is the climate change impact on PCB28 in the atmosphere as well (Figs. 9 and 10).

Even though the models projected significantly increased concentrations compared to the present-day scenario, the absolute concentrations by the end of the twenty-first century were several orders of magnitude below the present concentrations in all scenarios. Temperature and its impact on volatilisation of PCBs from both primary and secondary sources are the main driver for the model results. The impact of climate change versus the reduction of primary emissions can be considered minor. Environmental degradation of PCB and especially the international legislations and bans on PCB production and usage are of major importance for decreased future PCB concentrations.

#### MPI-MCTM

Cycling of PCBs in a changing climate (A1B scenario of the IPCC AR4) was simulated using a multi-compartment chemistry-transport model which is based on a coupled atmosphere-ocean model (MPI-MCTM) (Guglielmo et al. 2009). According to the MPI-MCTM model, the effect of the changing climate on PCB is enhanced volatilisation from ice-free surface seawater but also enhanced storage of the compounds in the areas of the Arctic Ocean that are covered by ice, except in the Laptev Sea (Fig. 13). The contribution of precipitation to the substance cycling will also increase. The total environmental residence time (i.e. persistence; *τ*_ov_) of PCB153 will be reduced by 40% in the 2090s compared to the 1990s, mostly due to increased biodegradation in soil and water. However, due to shifting distribution towards soil and land ice, *τ*_ov_ of the lighter PCB28 will increase in the Arctic. In general, the climate change effect on PCB concentrations in soil, air and sea ice causes an increase, which, however, is by far smaller than the effect of decreasing primary emissions. Meridional long-range atmospheric transport of PCBs into the Arctic will continue to decline in this century, but the decline rate will level off (Octaviani et al. 2015).

#### DEHM

The DEHM was used to model the fate and transport of PCBs in the environment (Hansen et al. 2008). Two decades were compared in the forecast: 1990–1999 as a starting point and 2090–2099 as the ‘end point’ and climate data were taken from a model run of ECHAM5-MPI-OM simulating the SRES A1B scenario of IPCC AR4 (Semazzi 2003; Winton 2006). The initial conditions for the two time periods that were compared were the same and the PCB emissions were assumed to be identical to get a clear signal from the impact of climate change without changing emissions as an additional factor. Further details on model parameters and input are described in Hansen et al. (2008) and Hansen et al. (2015).

**Fig. 14:**
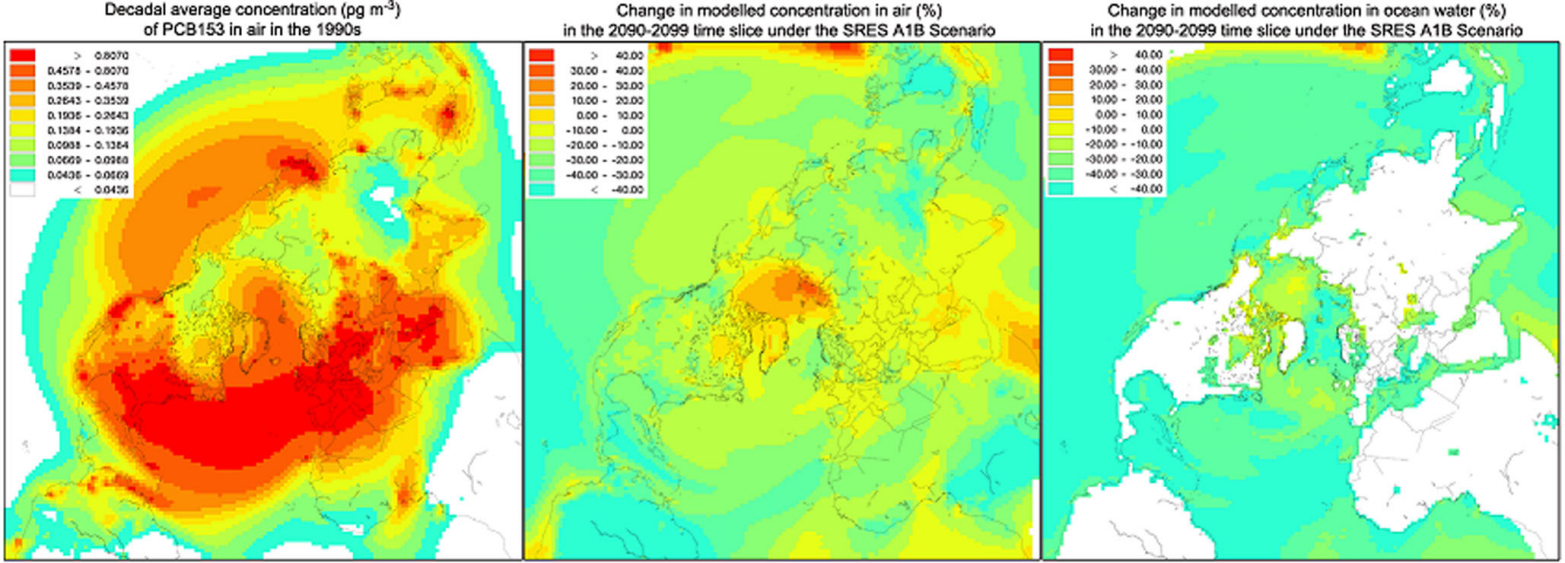
Modelled decadal averaged atmospheric concentrations of PCB153 in the 1990s (left), the change in modelled concentrations in the 2090–2099 time slice under the SRES A1B climate scenario in air (middle) and in ocean water (right)

For PCBs with two to five chlorine atoms, including PCB28 and PCB101, modelled concentrations in the 2090–2099 time period in Arctic air were similar to or slightly lower in the atmosphere in comparison to the 1990–1999 time period and modelled concentrations in Arctic Ocean water and soils were lower by 20–40%. For higher chlorinated PCBs such as PCB153, modelled concentrations in Arctic air were higher by about 5% in the 2090–2099 time period (Fig. 14 middle), concentrations in Arctic Ocean water were lower by about 40% (Fig. 14 right) and concentrations in Arctic soils were close to identical (not shown). For the highest-chlorinated PCB congeners that were considered (PCB180 and PCB194), modelled concentrations in Arctic air were 15% higher in the 2090–2099 time period, modelled concentrations in Arctic Ocean water were 10% lower and modelled concentrations in Arctic soils were slightly higher.

#### ECHAM5-MPIOM

**Fig. 15:**
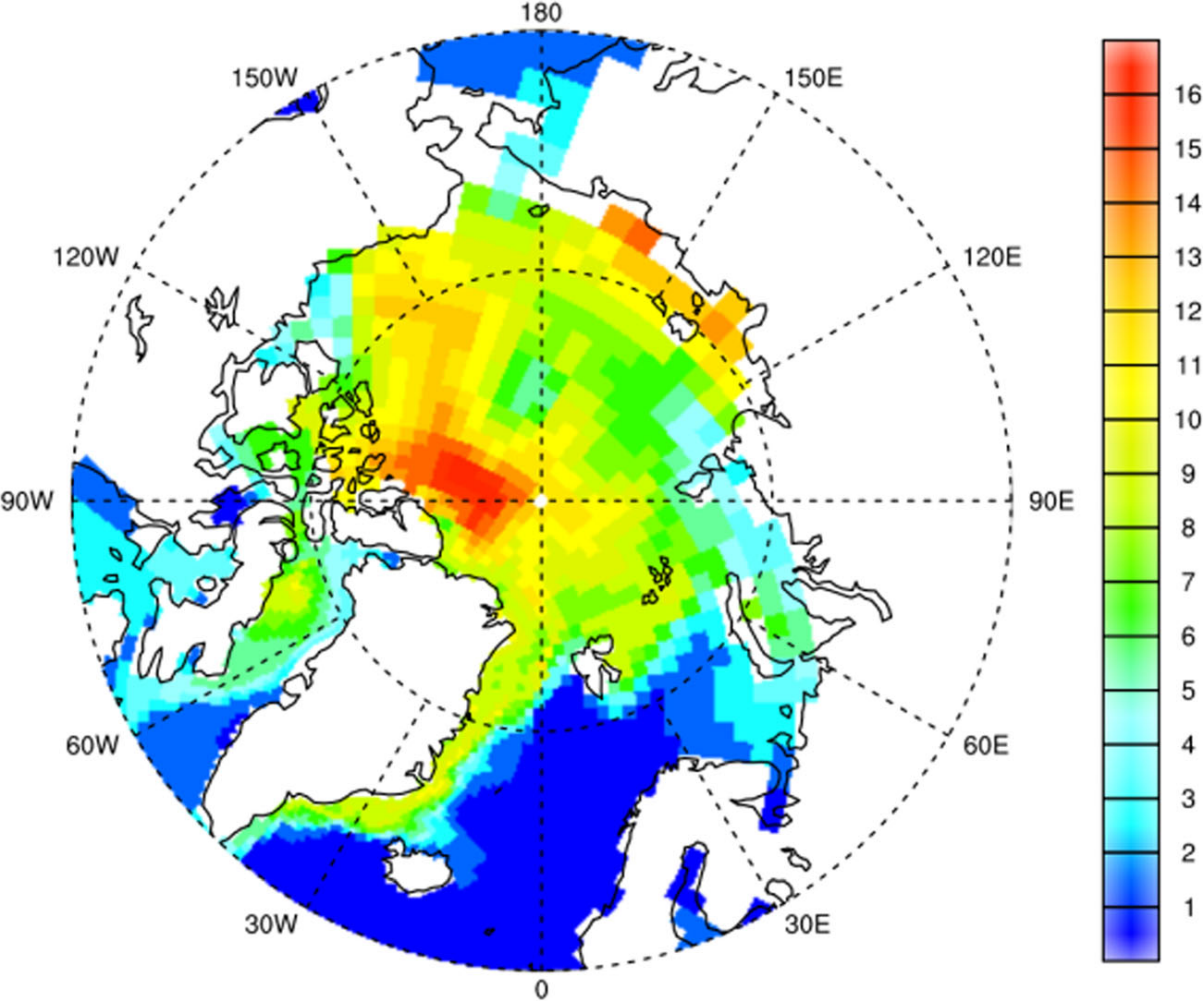
Present-day (annual mean of 2010) PCB153 mixing ratio in suspended organic phases, i.e. sum of dissolved and particulate organic carbon, and bulk phytoplankton and zooplankton (ng PCB/gC) in the Arctic Ocean

Simulations were made using a multi-compartment chemistry-transport model which is consists of one general circulation model for the atmosphere coupled to an ocean general circulation model (ECHAM5-MPIOM). This model also includes an ocean-biogeochemistry sub-model (Guglielmo et al. 2012; Hofmann et al. 2012; Stemmler and Lammel 2012). The results indicate that for the A1B scenario of the IPCC AR4, more PCB153 will be associated with the particulate organic matter in water *c*_POC_, in particular the colloidal mass (‘DOC’) in the multi-phase seawater system. Therefore, bioavailability of this congener at the bottom of the marine food chain is expected to increase (Fig. 15).

#### Modelling output from the *ArcRisk* project

All modelling tools used in the *ArcRisk* project agree upon most of the expected impacts of climate change on PCB concentrations in the Arctic. Their projected future (climate change scenario, neglecting emission reductions) concentrations are about a factor of 2 of relative increase compared to the baseline situation (today). The overall conclusion from the *ArcRisk* modelling studies is that the modelled concentrations of low-chlorinated PCBs in Arctic air are not as sensitive to climate change impact as the mid- and high-chlorinated PCBs are. Higher relative concentrations are expected for the mid-higher chlorinated PCBs under a climate change scenario (Lamon et al. 2009; MacLeod et al. 2011; Wöhrnschimmel et al. 2013).

Concentrations of PCBs in Arctic Ocean water are also higher under a climate change scenario according to BETR Research and MPI-MCTM results. In model experiments with DEHM that assumed the same emissions but different climate scenarios, modelled concentrations of PCBs in Arctic Ocean water were lower compared to BETR Research and MPI-MCTM projections under the climate change scenario.

The models BETR Research and MPI-MCTM suggest a similar role of global climate change on the atmospheric concentrations of highly chlorinated PCBs, e.g. PCB153, in both the Arctic and in the Baltic Sea region. The model results suggest increases in the atmospheric concentrations of these contaminants in the two regions with climate change compared to the present-day. The DEHM models forecast lower concentrations of high-chlorinated PCBs in seawater in the two regions, which is in agreement with the forecast of the multimedia chemical fate model POPCYCLING-Baltic that was adapted for the Baltic area (Kong et al. 2014). The pattern of Arctic Ocean water pollution is more heterogeneous in the MPI-MCTM simulation (Fig. 13) compared to DEHM.

## Biota exposure and PCB uptake in a warmer Arctic

Processes governing bioaccumulation are temperature-dependent. In addition to the physical-chemical and biotransformation properties of PCB climate change will inevitably affect the velocity and environmental stability of environmental pollutants (Walters et al. 2016). Thus, changes in magnitude of relevance for bioavailability can be assumed for several climate change scenarios. In a changing Arctic environment, food web structures (including composition, availability of prey, etc.) are expected to change and these changes within environmental processes will impact the environmental fate of pollutants in the Arctic ecosystem (Boonstra 2004; Fisk et al. 2001b; Hallanger et al. 2011a, b; Kallenborn et al. 2012; Riget et al. 2013).

### Bioavailability

Bioavailability can be defined in two distinct ways according to an earlier comprehensive report (Gobas and Morrison 2000):

1. The fraction of the total concentrations, in a specific medium or matrix (e.g. water and sediment) that can be absorbed by the organisms via a specific route of uptake
2. The rate or the extent to which a chemical is absorbed and accumulated by the organism

The *ArcRisk* work was mainly related to point (1). Bioavailability according to point 1 is largely controlled by the distribution of POPs between the phases of environmental matrices. For example, a POP’s bioavailability in water would be controlled by the distribution between the dissolved, particulate and dissolved organic matter phases. This distribution would be influenced by the physical-chemical properties (e.g. the octanol-water partition coefficient, K_OW_) of the PCBs, the particulate concentration in the water column, the properties of the particulate matter phases (e.g. organic matter content) and temperature. Temperature changes resulting from global climate change would therefore have a direct influence on partitioning of POPs in natural waters. For example, in the Baltic Sea, the equilibrium partitioning of hydrophobic organic contaminants between the particulate and dissolved phases in water decreased by a factor of five with a temperature increase of 20 °C (Smith and McLachlan 2006).

Borgå et al. (2010) used an aquatic bioaccumulation model to simulate the effect of global climate change on POP bioaccumulation in an Arctic marine pelagic food web. In this modelling approach, it was assumed that climate change would result in increasing primary productivity in the Arctic Ocean, resulting in increasing concentrations of particulate organic carbon (POC) in the water column. Borgå et al. (2010) also considered the effects of temperature on changing respiration, consumption and growth rates of species in the food web studied. Three chemicals were considered: γ-HCH, PCB52 and PCB153. In each case, a decrease in bioaccumulation was predicted because of global climate change. In the top-predator cod (*G. morhua*), these changes ranged from being negligible for γ-HCH to a 50% decrease for PCB153. These decreases were primarily controlled by reduced bioavailability resulting from dilution of the chemical in the larger mass of POC due to the assumed increase in primary productivity. Therefore, the effect of increased temperature on partitioning discussed above was offset by the increase in POC. It should be noted that simulating how global climate change will affect future primary productivity in the oceans is highly uncertain (Cousins et al. 2011a, b). For example, contrary to the assumption of Borgå et al. (2010), Boyce et al. (2010) observed a decrease in primary productivity in the Arctic Ocean and associated this decrease to limited nutrient supply caused by temperature-driven stratification of the surface oceans.

We conclude from the above discussion that the major impact of climate change on bioavailability is likely due to changes in distribution between exposure media due to increasing temperature or changes in the primary productivity as opposed to the direct bioenergetic impacts of temperature on PCB uptake through changes in food consumption or metabolic rate. Large uncertainties remain, however, concerning how primary productivity in the world’s oceans will be affected by global climate change. This information was developed in *ArcRisk* and incorporated into models and experiments during the project.

#### Food web transfer variations in a changing Arctic climate

Climate change can impact environmental processes and the transfer pathways of POPs within the food web such as bioavailability (as discussed above), metabolism and trophic structure. Cousins et al. (2011a, b) and Gouin et al. (2013) have reviewed the impact of climate change on bioaccumulation of POPs in food webs, and their main conclusions were that ‘indirect’ effects (e.g. changes in human diets, species distribution and primary productivity) are likely to be of higher importance for humans and the environment compared to ‘direct’ effects (bioenergetic processes, such as consumption rate, metabolism and growth). However, indirect effects are much more difficult to include in models as well as in empirical studies. Hence, we need better and more thorough understanding of trophic interactions and changes within the food web to fully understand the impact of climate change on bioaccumulation of PCBs in the future.

## Contemporary and future human exposure scenarios

PCBs and other POPs may enter humans via air, food or through contact with the skin (Cao et al. 2014; Linares et al. 2010; Lorber 2008; Turyk et al. 2009). Among these entry routes, the diet is the major source of PCBs, especially fatty fish, meat and dairy products. PCBs have been detected in a suite of body tissues and fluids such as maternal and children’s blood and/or serum, cord blood, foetal adipose tissue, placenta, infant blood, blood from males and breast milk in Arctic peoples (AMAP 2015; Nøst et al. 2013; Bonefeld-Jørgensen 2004; Donaldson et al. 2010; Dudarev et al. 2004; Hansen 2000; Klopov et al. 1998; Nøst et al. 2013). In the blood of breast-fed infants, the concentration may be many times higher than in maternal blood (Boucher et al. 2010). PCBs have a long half-life in the body and by fitting a population-level pharmacokinetic model to biomonitoring data for human blood, half-life estimates of 15.5 years for PCB170, 14.4 years for PCB153 and 11.5 years for PCB180 were estimated (Ritter et al. 2011). PCBs that are not bio-transformed are only slowly excreted, mainly through the faeces, urine and breast milk. Throughout the past few decades, a variety of more or less subtle effect endpoints such as activation of the aryl-hydrocarbon receptor (AhR) and the pregnane X receptor (PXR), foetus development and mental development among children have been identified in the literature, especially based on Arctic studies (Abass et al. 2013; Bonefeld-Jørgensen 2004; Deutch et al. 2007; Donaldson et al. 2010; Hansen 1998, 2000; Hansen et al. 2002; Hansen et al. 2010; Klopov et al. 1998; Letcher et al. 2010; Odland and Nieboer 2012).

### Toxicokinetic modelling and future risk predications

Several earlier trend studies report PCB levels in Arctic indigenous populations (Donaldson et al. 2013; Dudarev 2012; Gibson et al. 2016; Kruger et al. 2012; Ryan and Rawn 2014; Schaebel et al. 2017; Singh and Chan 2017; Valera et al. 2013a, b; Veyhe et al. 2015).

**Fig. 16:**
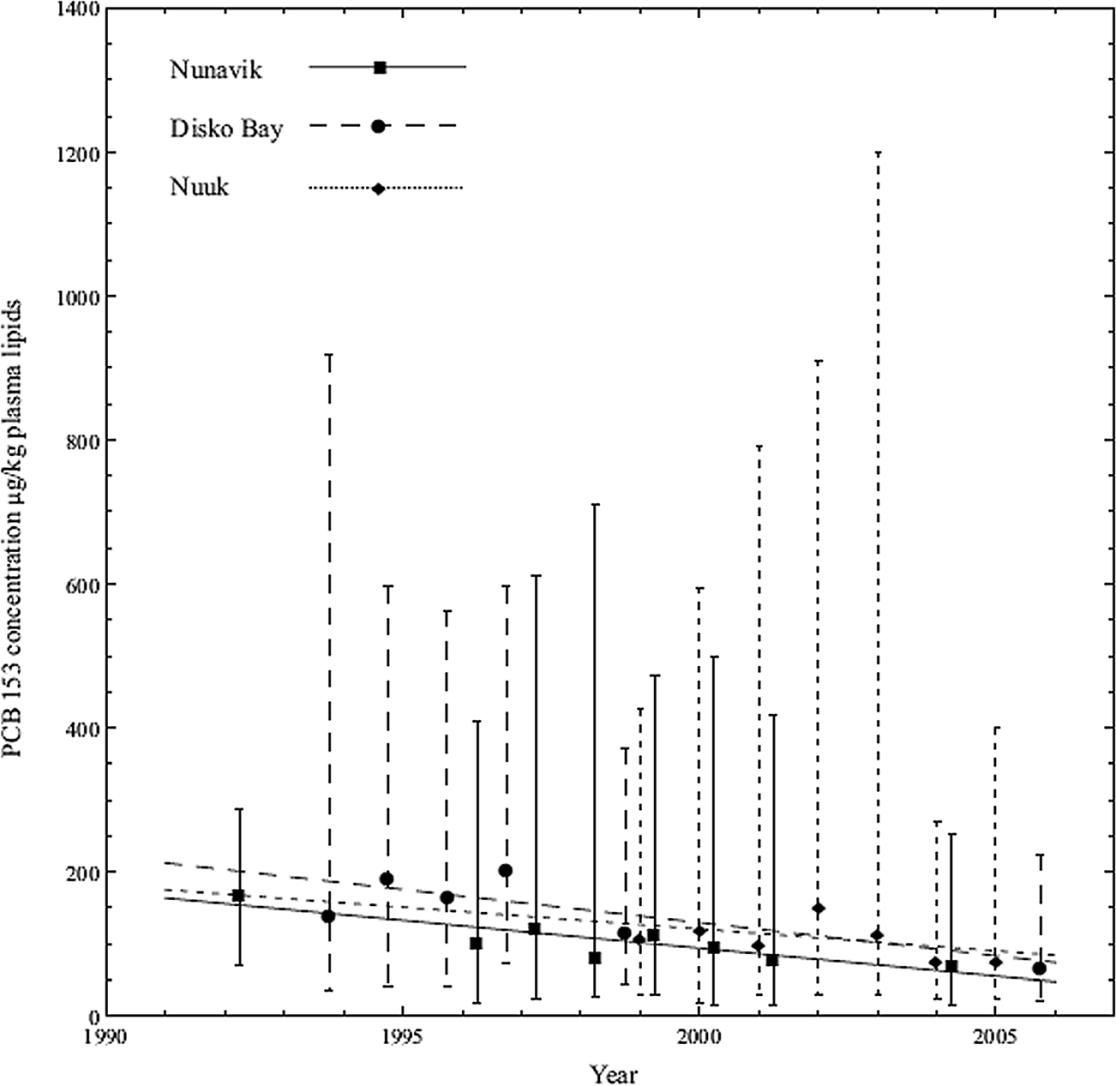
PCB153 concentration trends (geometric mean and range) in plasma lipid (μg/kg) among pregnant women from Disko Bay (Greenland), Nuuk (Greenland) and Nunavik (Quebec, Canada) in the period 1992–2007. Reprinted (Abass et al. 2013) with permission of Elsevier

PCB153 is among the most prevalent PCB congener found in human populations and as such has been one of the indicator PCBs for monitoring in biota (Fisk et al. 2005; Gewurtz et al. 2006; Gomez-Ramirez et al. 2014; Kalinovich et al. 2008; Letcher et al. 2010; Wolkers et al. 2006; Xu et al. 2013). The trend of geometric mean concentration (declining for all three locations) of PCB153 in plasma lipid from pregnant women during the years 1992–2007 in Inuit women from Disko Bay and Nuuk (Greenland) and Nunavik (Quebec, Canada) is shown in Fig. 16. The decreased concentrations of PCB153 in pregnant women from the Disko Bay area may be due to a decrease in the consumption of traditional food originating from animals at high trophic levels. There has clearly been a trend for decreased exposure to PCB153 in Arctic. The lower concentrations in Nuuk women compared to Disko Bay are most likely due to lower consumption of traditional diet that is rich in marine mammals in Nuuk (largest town in Greenland) compared to the more rural Disko Bay. The geometric mean concentration of PCB153 (Fig. 16) measured in plasma lipid has decreased from 111 to 172 μg/kg plasma lipids during the 1990s to 40–79 μg/kg plasma lipids during early 2000s at all three locations (Abass et al. 2013). The analyses of trends and data continue after the ArcRisk work as well and can be found in the recent AMAP assessment (AMAP 2015, p. 2015).

**Fig. 17:**
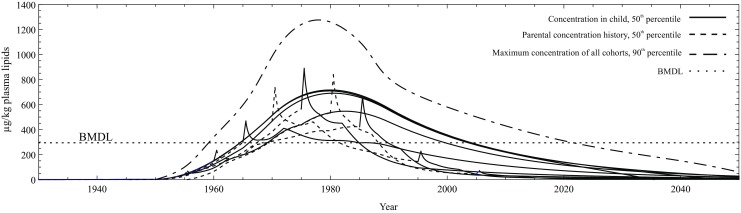
Extrapolated concentrations of PCB153 (μg/kg plasma lipids) in pregnant Inuit women from Nunavik, Disko Bay and Nuuk for different birth cohorts of 1940, 1950, 1960, 1970, 1980 and 1990. The estimated concentrations of PCB153 in plasma lipids for the 50th and 90th population percentiles of birth cohorts are given and shown. Both estimates, the dotted and solid curve for 50th population percentile while dashed envelope estimates maximum concentrations of all birth cohorts for 90th of population percentile. They are based on the curves of reference daily intake presented in Figs. 18 and 19. The health risk of PCB153 is estimated by using a benchmark dose level (BMDL) of 300 as a toxicological cutoff point. Reprinted (Abass et al. 2013) with permission of Elsevier

PCB153-associated human health effects and risks were assessed using data obtained from the AMAP biomonitoring programme presented above and a one-compartment population-based pharmacokinetic model. The aim within *ArcRisk* was to extrapolate body burden and exposure to the whole lifespan of the population. The results of the modelled body burden are presented in Fig. 17.

**Fig. 18:**
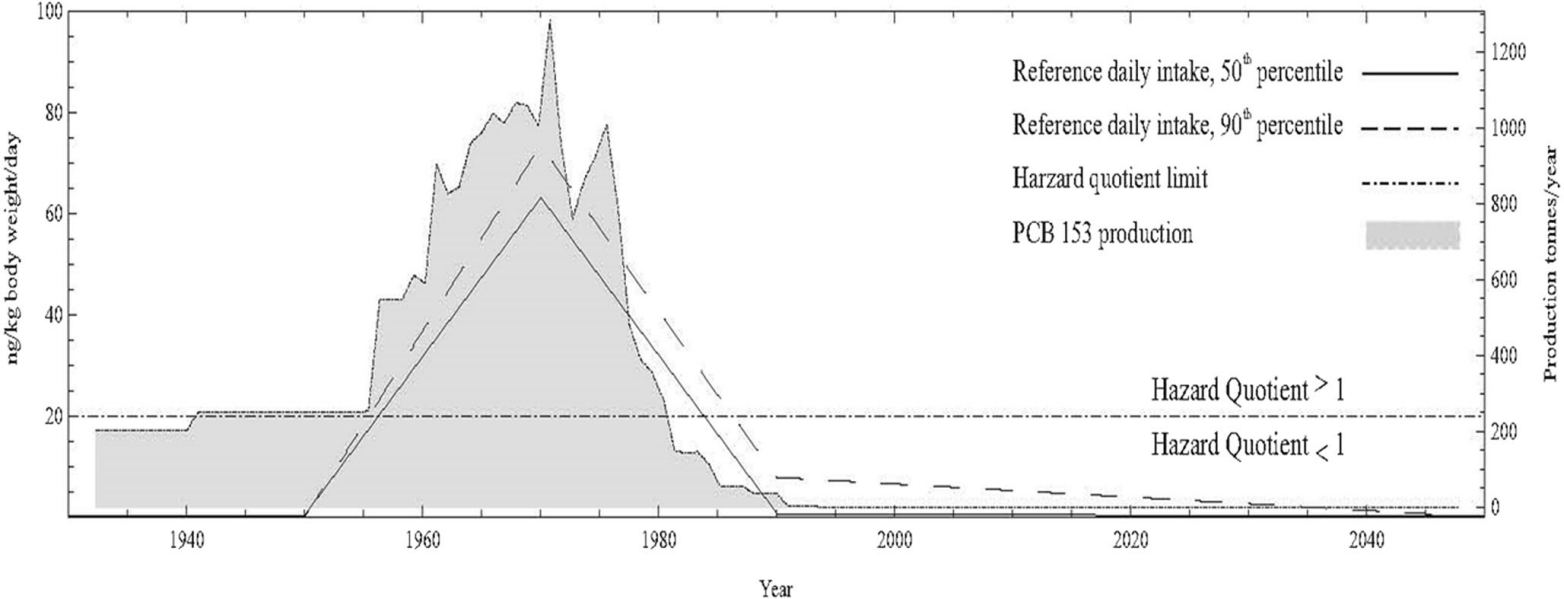
Modelled reference daily intake (ng/kg-bw/day) of PCB153 for an adult (50th and 90th) and production trend of PCB153. Reprinted (Abass et al. 2013) with permission of Elsevier

**Fig. 19:**
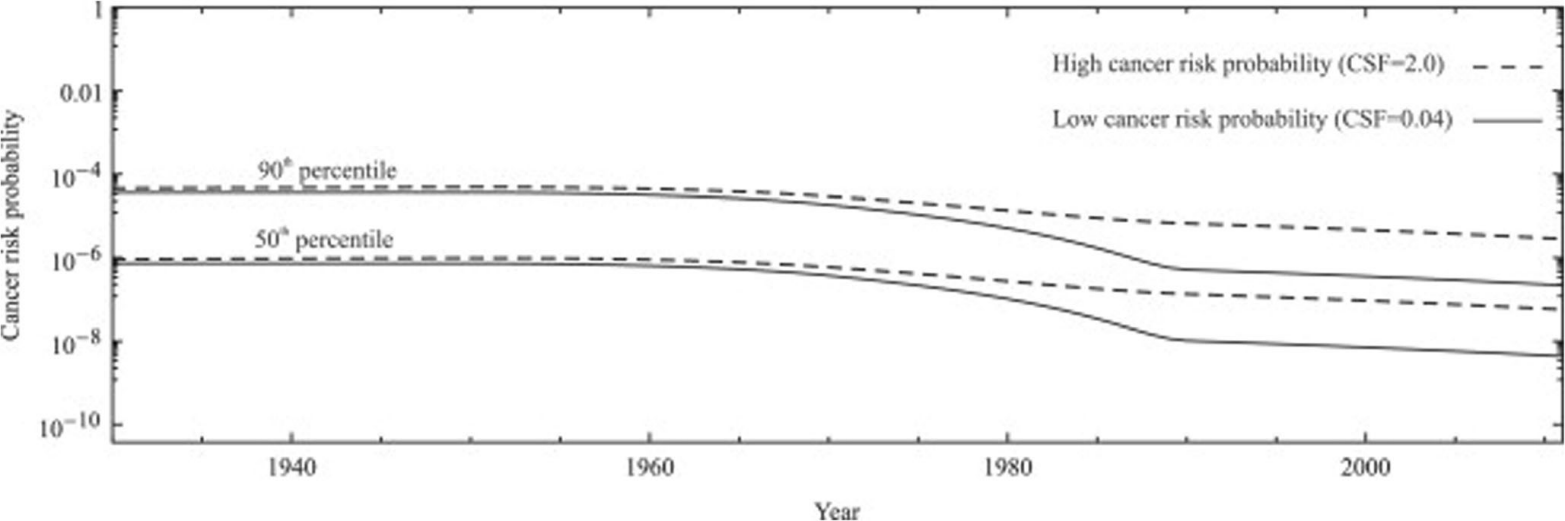
Cancer risk probability estimates for the high (cancer slope factor, CSF = 2.0) and low (CSF = 0.04) slope factors for the 90th and the 50th percentiles of the birth cohorts. The year-axis indicates the time of birth of the cohort. Reprinted (Abass et al. 2013) with permission of Elsevier

A hazard quotient (HQ) is the average daily dose (ADD) divided by a reference dose (RfD) and gives an estimate of non-cancer related effects. Abass et al. (2013) used HQ to estimate the exposure to PCB153 (Fig. 18) during recent decades for Arctic human populations. The 90th population percentile during the years 1955–1987 and the 50th population percentile during 1956–1984 had HQ > 1, which means that the exposure may cause a potential adverse, non-cancer health effect. Cancer risk related to PCB153 was also estimated within the same study and the range for the 90th percentile was from 4.6 × 10^−5^ to 1.8 × 10^−6^ between 1930 and 2049 (Fig. 19). Further details on methods and results are described in Abass et al. (2013). This kind of toxicokinetic modelling in combination with the United States Environmental Protection Agency Integrated Risk Information System (US-EPA-IRIS) risk assessment framework proved to be very useful for prediction and assessments of human health risks related to POPs.

### PCBs in Arctic and European populations

**Fig. 20:**
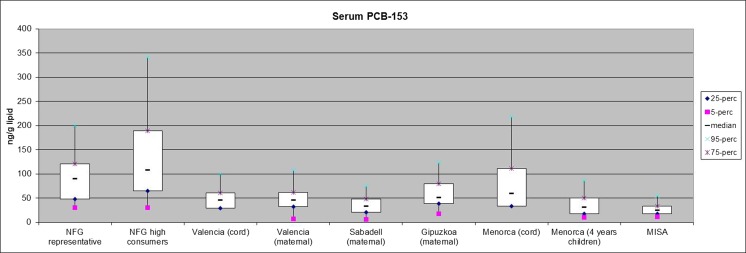
Overview of serum PCB 153 concentration in *ArcRisk* study groups. NFG = Norwegian Fish and Game study, MISA = Northern Norway Mother-and-Child Contaminant Cohort Study

As a part of the ArcRisk project, a survey including both Arctic (Norway) and European cohorts (Spain) was conducted and the results for PCB153 in blood serum are shown in Fig. 20. The highest exposure was found among participants in the Norwegian Fish and Game (NFG) study, while participants in the Spanish INfancia y Medio Ambiente: Environment and Childhood (INMA) and the Northern Norway Mother-and-Child Contaminant Cohort Study (Norwegian MISA) studies had the lowest exposure. However, the participants in the NFG study were older (median, 55 years; range, 21–80 years) than the participants in the other studies and, hence, higher concentrations could be expected. Furthermore, participants in the NFG high consumer group were invited due to high consumption of food that generally contains higher levels of POPs compared to other food items. Human PCB exposure is decreasing in several regions and hence the sampling year should be noticed (Donaldson et al. 2010). The NFG study samples were from 2003, while the sampling period and ages are more comparable between birth cohort mothers from Spain (INMA, sampling years 2004–2008, except Menorca which was sampled 1997–1999) and Arctic Norway (MISA, sampling years 2007–2009). The mean and upper range of exposure (both in maternal and cord blood) was higher among the INMA participants compared to the MISA cohorts. Thus, this first comparison indicates that women of fertile age living in Spain have higher PCB exposure than women in the northern part of Norway (Brorström-Lundén et al. 2013; Fernandez et al. 2007; Guxens et al. 2012; Llop et al. 2017; Morales et al. 2013).

**Fig. 21:**
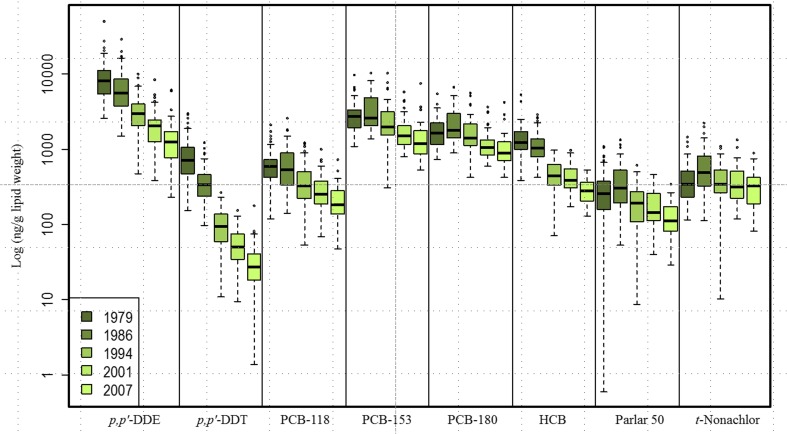
Concentrations (ng/g lipid, *y*-axis: log scale) of selected POPs analysed in repeated serum samples of men (*N* = 51, 51, 45, 48 and 52 in 1979, 1986, 1994, 2001 and 2007, respectively) from Northern Norway. Parlar 50 represents toxaphenes and *t*-nonachlor the chlordanes. Boxes extend from the 25th to the 75th percentile, horizontal bars represent the median and whiskers extend 1.5 times the length of the interquartile range (IQR) above and below the 75th and 25th percentiles, respectively; any outliers are represented as points. *p < 0.05 and **p < 0.001 for comparisons between pairs of consecutive sampling years. Please note the figure is reproduced from Nøst et al. (2013)

A study on POP distribution in the very same Norwegian men sampled from 1979 to 2007 (Nøst et al. 2013) showed declining concentrations for all POPs (Fig. 21) except for chlordanes (e.g. *trans-*nonachlor). Decreasing trends were observed from 1979 and onwards in concentrations of most penta- and hexa-chlorinated PCBs. On the contrary, hepta-chlorinated PCBs increased from 1979 to 1986, before they began to decline (Fig. 21). Nøst et al. (2013) showed that the POP concentrations decreased during 1979–2007 in men from Northern Norway and that the average ΣPOP concentrations in 2007 were one third of the concentrations measured in 1979. The years 1979 and 1986 had the peak PCB153 concentrations, which confirm that this period was the one with the highest human exposure. The decreasing trends in serum concentrations likely reflect declining environmental concentrations due to reduced emissions during the same time period.

### Scenarios of future of human exposure and potential health effects of PCBs in the Arctic

Risk assessment studies of pollution-related health effects in Arctic populations are usually conducted using long-term retrospective epidemiological studies. Even though there is a lot of existing information on the topic, few studies report relationships between POP levels and human health or health-related endpoints. Hence, *ArcRisk* therefore conducted a comprehensive meta-data analysis of results from the Arctic population studies on health effects. Three systematic review articles (Candolin et al. 2014; Nieminen et al. 2013a; Nieminen et al. 2013b) on the association between PCB exposure levels and secondary sex ratios identified the following limitations with respect to meta-analysis on health effects of PCBs:

1. The number of relevant articles with epidemiological data is very limited.
2. The findings of epidemiological studies are analysed and reported in ways that are often not comparable.
3. Results across repeated studies of the same phenomena are rarely identical due to reasons that include differences in analytical methods and genetic differences between the populations studied.
4. Different statistical methods are used in different studies and publications even though their main aims are identical, e.g. investigations of the relationship between PCB exposure levels and health outcomes.
5. The quality of method descriptions also varies: detailed descriptive statistics of the variables included and standard error for regression coefficients and/or the mean differences were not always reported.

## Summary and conclusions

The *ArcRisk* research team conducted research on long-range transport, occurrence and fate, exposure and health impacts of selected contaminants in Europe and the Arctic during a 4-year period (2009–2013). The *ArcRisk* research, combined with results from complementary studies, contributed significantly to the current scientific understanding on the influence of climate change on PCB/pollutant distribution, cycling and effects in the Arctic. The combined results from models, measurements and studies on health effects have been compiled to provide an overall picture of PCBs and to describe the links between emissions and health effects in the Arctic. This review presents a comprehensive overview on PCBs that shows our current understanding of the complex relationships between sources, transport, bioaccumulation, exposure and health impacts of PCBs in relation to climate change, especially for Arctic environment and human populations.

The most relevant climate parameters that impact transport pathways of PCBs to the Arctic and environmental processes in the Arctic environment are changes in temperature, precipitation, sea and land ice cover and the global circulation of the atmosphere and the oceans.

### Emissions

Emissions of PCBs still occur from, e.g. buildings and waste dumps (e.g. primary sources), although at decreasing rates. Secondary emissions of PCBs accumulated in environmental reservoirs (sediment, water, soil, snow and ice) are becoming more important than primary sources. Climate change will most likely increase primary and secondary emissions relative to levels that would be expected under current conditions but not to an extent that the overall decreasing trend of PCBs in the remote environment will be confounded.

### Important pathways to the Arctic

Atmospheric long-range transport is a major route for the global distribution of PCBs to the Polar Regions and deposition from the atmosphere is an important pathway of PCBs to both terrestrial and marine environments in the Arctic.

In addition to long-range transport via the atmosphere, transport via oceanic currents is also important for the occurrence of PCBs in the Arctic. Climate change is likely to affect all these pathways and their subsequent environmental fate.

The legislation covering PCBs that began in several countries during the 1970s has had the desired effect. However, with the large quantities of PCBs used and their persistence, we still see ongoing emissions of PCBs, and hence they are present in biotic and abiotic matrices all over the world. Lessons learnt from studies of trends of PCBs confirm that legislation in combination with long-term monitoring remains a very important tool for decreasing the environmental and human health-related risks associated with persistent, toxic and bioaccumulating compounds.

### Key processes

Precipitation was identified as an important factor for PCBs present in surface compartments such as the seasonal and perennial snow pack (the latter associated with ice caps for example). Climate-related effects such as changes in precipitation patterns and erratic and longer snow-melt periods will influence the accumulation and release of PCBs from cryospheric compartments like snow, firn and ice.

PCBs can accumulate in young/single-season sea ice thus providing a chemical stock for surface marine waters although it is unclear how the changing nature of sea ice—its composition (first-year ice vs multi-year ice) and areal cover—will affect the pathways and mobility of PCBs in surface marine waters. During pronounced seasonal thaw, accumulated contaminants like PCBs will be released to the water thus providing a focused exposure mechanism for ice-associated organisms at the base of the marine food web.

In total, PCB releases from sea ice and snow do not provide large inputs to the Arctic oceans in comparison to those forecast for the major pan-Arctic rivers that drain into coastal Arctic seas.

### Impact of climate changes on PCBs in the Arctic

The outcome from the models used in *ArcRisk* indicated that PCB concentrations will show a relative increase in the Arctic Ocean and atmosphere, mostly due to the climate change impact on temperature, which will affect the volatilisation of PCBs from both primary and secondary emission sources. Atmospheric PCB concentrations have been decreasing in the Arctic during the last few decades. Although some of the modelled concentrations of PCBs were higher under the climate change scenarios, the absolute concentrations are forecast to be several orders of magnitude below present levels in all scenarios by the end of the twenty-first century. This is directly linked to the long-term reductions in primary emissions.

It is important to remember that the models do not include all factors that may influence future PCB emissions/transport. Increased transport (shipping, air traffic, etc.) and tourism in the Arctic and changes in diet among indigenous peoples are all examples of factors that can be of high importance for assessing the future or changing impact of PCBs on human health and the Arctic environment, but these factors are extremely difficult to incorporate into a model. Climate change modelling is complex and indirect effects such as changes in vegetation cover, animal distribution and land usage are not included in the total assessment of PCB behaviour. Nevertheless, *ArcRisk* has helped to fill knowledge gaps as well as identify research areas where knowledge on the impact of environmental pollutants on human and ecosystem health in the Arctic in a changing climate is needed.

### Bioavailability, food web transfer and concentrations in biota

One of the most important factors that climate change affects is the temperature, which is a driver for several other environmental processes, including partitioning of PCBs between air, water, soil and biota. Hence, climate change may cause changes in PCB distribution and thereby exposure pathways for biota, e.g. impacts on primary production, which may change the amounts of particulate or dissolved organic material (POM/DOM).

### Human health

PCB concentrations will decrease over time in humans, mainly owing to implementation of international legislation. There are still knowledge gaps on the risk assessment of PCBs on human health due to the relatively small number of studies and difficulties in comparing among studies, although meta-analysis studies can provide good overviews and data for these risk assessments. For indigenous peoples, changes in diet associated with increased consumption of imported processed foods as well as switches to different species depending on seasonal abundance and climate-induced changes in species composition are likely to have the most marked effect on PCB exposure in the near future.
